# Challenges to conservation: land use change and local participation in the Al Reem Biosphere Reserve, West Qatar

**DOI:** 10.1186/1746-4269-6-28

**Published:** 2010-10-21

**Authors:** Paul Sillitoe, Ali A Alshawi, Abdul K Al-Amir Hassan

**Affiliations:** 1 Qatar University, Doha, Qatar; 2 Durham University, Durham, UK

## Abstract

One response to humanity's unsustainable use of natural resources and consequent degradation, even destruction of the environment, is to establish conservation areas to protect Nature and preserve biodiversity at least in selected regions. In Qatar, the government has shown strong support for this approach, confronted by the environmental consequences of oil and gas extraction and rapid urban development, by designating about one-tenth of the country a conservation area. Located in the west of the peninsula, it comprises the Al Reem Reserve, subsequently declared a UNESCO Biosphere Reserve. Several approaches have figured in conservation, currently popular is co-management featuring participation of the local population, which recognises that people's activities often contribute to today's environment, with the promotion of bio-cultural diversity. However, these assumptions may not hold where rapid social and cultural change occurs, as in Qatar. We explore the implications of such change, notably in land use. We detail changes resulting with the move from nomadic to sedentary lifestyles: in land access, which now features tribal-state control, and herding strategies, which now feature migrant labour and depend on imported fodder and water, underwritten by the country's large gas and oil revenues. Current stocking arrangements - animals herded in much smaller areas than previously - are thought responsible for the degradation of natural resources. The place of animals, notably camels, in Qatari life, has also changed greatly, possibly further promoting overstocking. Many local people disagree. What are the implications of such changes for the participatory co-management of conservation areas? Do they imply turning the clock back to centrally managed approaches that seek to control access and local activities?

## Overview

Degradation of the natural environment and need for conservation measures are urgent concerns with ever more evidence of human activities despoiling the planet, exacerbated by current climate change predictions. The consequences are particularly graphic in marginal and harsh environments such as the deserts of the Middle East, where some regions, which appear denuded of plant and animal life, can look to the outsider like barren moonscapes. It is widely agreed that we need biodiversity conservation [1-3]. The assumption behind such initiatives is that the environment in selected areas needs protection -- from human activities in particular -- to prevent irreparable damage occurring to natural habitats and possible loss of species; and is considered particularly urgent in places rich in biodiversity or exemplary examples of certain ecosystems [1,4,5].

**Figure 1:**
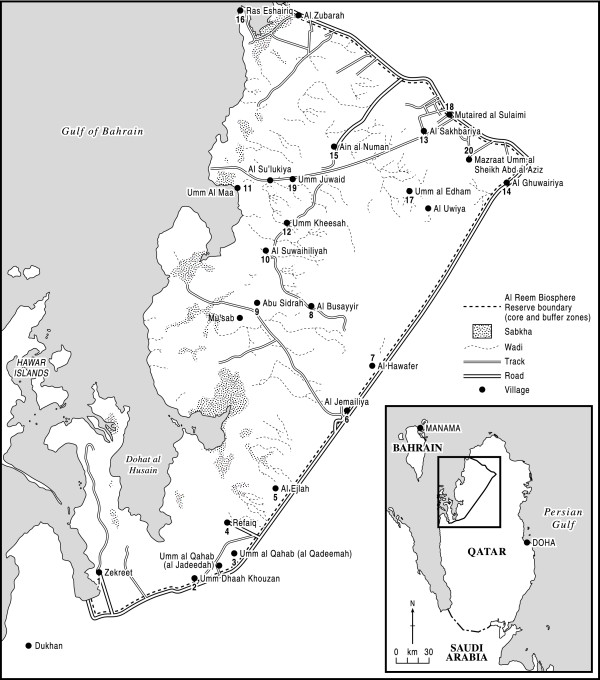
**Al Reem region**.

The Government of Qatar has shown a strong commitment to conservation in its 2030 National Vision [6], where under the fourth development pillar, concerning the environment, it says that the State seeks 'to preserve and protect its unique environment and nurture the abundance of nature granted by God'. It has signalled the seriousness of its intent in declaring the Al Reem region, approximately 10% of Qatar's land area, a conservation reserve under the UNESCO Man and Biosphere [MAB] programme. The Reserve is situated in the north-west of the Qatar peninsula (see Figure 1); established by the Supreme Council for the Environment and Natural Reserves in 2005 - following declaration of its protected status by Emiri Decree 7 (2005) - it became a Biosphere in UNESCO's MAB programme in 2007 (UNESCO 2007 'Al-Reem Reserve: UNESCO MAB Biosphere Reserve Nomination File', submitted to The Supreme Council for the Environment and Natural Reserves, State of Qatar page 5), [7]. It lies within parts of both Jemailiya and Madinat Al Shamal Municipalities; the two towns of Jemailiya and Al Ghuwairiya are located on the highway that marks the Reserve's eastern boundary.

This paper casts a quizzical eye over current conservation thinking, which has moved from exclusion to participation, from advocating reserves that restrict human access and activities to co-management arrangements that incorporate local populations and their practices. The latter view accepts that humans are part of ecosystems and adopts a bio-cultural diversity perspective, which advocates that as peoples' activities influence any environment, so they should be included in any conservation regime. But what happens when those activities change and threaten contemporary ecological relations? By reviewing land use practices in Qatar, where dramatic and rapid social change has occurred, we query the assumptions of co-management that seek to use local knowledge as a conservation resource. While the Bedouin may identify themselves as the appropriate stewards of the desert that is their homeland, the changes that have occurred recently in their lives may compromise this claim, if not the possibility that their activities have been degrading resources over centuries. With the switch from a nomadic to sedentary lifestyle, animals are now herded in much smaller areas. It is widely thought that current stocking arrangements, nominally controlled by a system of government licensing, are responsible for the degradation of natural resources, featuring large herds managed by migrant labour and dependent on imported fodder and water.

The place of animals, notably camels, in Qatari life, has changed greatly. They are now symbols of social status and Arab identity rather than sources of livelihood. Current economic arrangements featuring large hydrocarbon revenues underwrite the resulting competitive overstocking. From a conservation of nature perspective, it looks as if we should go back to instituting exclusion zones. But this is politically implausible in Qatar and participation the only option. Indeed the Al Reem reserve already features an element of co-management in that rangers are local persons. But their understanding of issues regarding conservation is limited, as is that of the rest of the local populace, as a survey of awareness and attitudes to the reserve shows, with many people suspicious of unwelcome interference in and restrictions on their lives. Furthermore, they do not accept that their herding practices are harming the environment; they think any changes are climatic. The grand question is how to make participatory parks a reality in such contexts of rapid change. There is clearly a need for some new thinking, to navigate our way around such conundrums and promote a new sustainable accommodation between human population and environment.

### From prohibitive to participatory parks

A concern for conservation is not entirely new, albeit current events have heightened awareness. We find it mentioned in ancient scriptures; for example Mosaic Law forbids the destruction of fruit-bearing trees and the killing of birds tending nests (Deuteronomy 20:19 & 22:6). In contemporary times, with growing recognition of ecological damage following industrialisation, we have the establishment of national parks, and most recently biosphere reserves, to protect the environment and promote conservation. Such parks have a considerable history; for instance Yellowstone National Park in the USA, arguably the world's first, was established in 1872, followed in 1879 by the Royal National Park in Australia south of Sydney and the Rocky Mountain National Park in Canada in 1885 - [8]. From the start, these parks were seen as protected areas, which minimise human interference in the natural environment; after the so-called 'Yellowstone model' [9,10,4]. According to the International Union for the Conservation of Nature and Natural Resources (IUCN), for instance, a national park is a region where protection of nature takes precedence and the ecological environment is not materially altered by human occupation and exploitation--and steps are taken to prevent or stop such--with visitors entering under controlled conditions. But such measures to protect nature from human interference were only possible where ruthless colonialism displaced local populations.

Following the establishment of reserves in various parts of the world, a process that has burgeoned since the mid 20^th ^century, it has become apparent that the original idea of excluding humans from such areas leads to considerable problems, even conflicts [11-15]. This became evident with the establishment of national parks in heavily populated regions, such as parts of Europe; national parks in England, for instance (designated in 1949 - [16]), often include substantial human settlement and resource use, and the land remains largely in private ownership. In an attempt to reduce local resentment at the establishment of parks that interfere with previous land use, various schemes have been devised [17,18], such as the designation of zones that differ in access and permit human activities, from core zones where classic conservation measures apply and humans are largely excluded to conserve pristine nature through to buffer and transition zones where varying human activities are permitted that interfere in nature.

It was also realised that the activities of local people contributed to the current environment; often they managed aspects of it. In other words, humans are part of ecosystems and they have to be considered along with other animals that inhabit any region in thinking about conservation, their activities inevitably intervening in nature's arrangements [19,20]. In this event it makes no sense to exclude local people from parks [21]. Indeed it is questionable if there are many regions in the world that are truly wilderness as conceived by the pioneers of conservation because humans have occupied and manipulated most environments on Earth to some degree, even if hunter-gatherers [22]. It is arguable that appreciation of local practices will further conservation interventions in both ecologically and sociologically, as these often represent understanding rooted in highly sustainable adaptations [23]; for example Arabs have managed to live for centuries in delicate desert environments without apparently irreparably degrading their resources [24,25]:

"Over thousands of years of experience, pastoral nomads have devised effective means of predicting and reacting to changing environmental opportunities. Many of these people have created what social and ecological scientists would call "sustainable use" practices or "ethnoconservation" systems, making them ideal partners for modern conservation and development efforts" pages 785-86 [26].

The implication is not that all human activity has been environmentally benign, until recently. It is possible that some human induced environmental change has resulted in resource degradation of unknown extent for centuries; the truth is that we do not know much about the state of the desert historically. Nonetheless, the degradation was not serious enough to threaten traditional livelihoods. This has led to the emergence of the idea of bio-cultural diversity to indicate that culture may contribute to biological diversity, that human activities may support, not threaten biodiversity.

After repeated encounters with resentful local populations seeking to subvert the restrictions imposed on their previous use of land and natural resources [27], and the realisation that their activities contribute to the contemporary environment, the idea of co-management regimes emerged, which seek to involve local people in park management, utilising participatory methodologies that have emerged in development in the last three decades or so. It has become increasingly evident that successful biodiversity reserves seek to accommodate local knowledge and resource use practices as best they can and ideally include local people in formulating and operationalising their management strategies [28,29]. It is necessary to involve local people not only because they have knowledge relevant to making any reserve a success but also to ensure they contribute to the devising of the management regime, so that they subsequently sign up to it, as socio-culturally appropriate and complying with their knowledge and expectations [30-33]; excluding people does not work, as it fuels resentment [9,34,13,35].

While co-management regimes have overcome certain objections and problems, others remain, notably of a political hue, leading sometimes to further conflict. It is often a challenge to incorporate local aspirations, activities and knowledge in a way that does not conflict with the aims of conservation [36,29,5]. It has become increasingly evident in the last two decades, with attempts to incorporate local populations in the planning and management of parks, that different parties or stakeholders may have differing views as to how to proceed [14,37]. The conflicts may be both external and internal, that is between the local community and other parties (park authorities, policy makers and international agencies), and between different interest groups within the local community with political infighting, for instance between elites seeking to control the new source of power. A key aspect of winning local consent and co-operation is the ability to identify such potential conflict points and devise strategies to circumvent them in the interests of all stakeholders, and there is now a sizeable literature on reconciling differences between stakeholders [38-41].

The issues become increasingly complex where extensive social change occurs, such as has occurred in the Gulf [42-46]. It can result in confusion and discord, as persons differ in their understanding and interpretation of events [35,47]. The assumption that the protracted negotiations that may characterise the inclusion of different parties implicated in a conservation reserve are worth it - as local knowledge and practices have a long history and are well adapted to exploit a region's resources while maintaining ecosystem balance [48,49] - this assumption breaks down where rapid social change and economic development occur [50-52,25], leading to changes in resource and land use, so altering human-environment relations and the applicability of local knowledge. The cultural dimension that features in the bio-cultural diversity formulation changes to such an extent that human management and/or interference in the environment becomes potentially destructive from a conservation perspective. The formulation of a reserve's management strategy becomes considerably more complex, as all parties have to learn what may be an ecologically sustainable adaptation under the changed conditions (D. Chatty n.d. Adapting to Biodiversity Conservation: The Mobile Pastoral Harasiis Tribe of Oman. Unpub. Typescript). This is the position currently in the Al Reem region, where such extensive social change is starkly evident. Similarly in the Jubail Reserve of neighbouring Saudi Arabia, where "Present human activities ... for the most part a continuation of traditional practices of long standing which may have done little damage in the past but which have now become unsustainable due to the technological, economic and demographic changes of recent decades" page 491 [53].

## Methodology

The Al Reem study featured standard anthropological/sociological methods, namely observation, informal interviews and formal surveys. The project started out as a socio-economic review of local communities in the region (P. Sillitoe, Al-Shawi, A., Al-Amir Hassan, A. K. and Abdel-Hafiz, M. 2009 Socio-economic survey report for Qatar Shell proposed biodiversity offset investment at Al Reem Biosphere Reserve. Submitted to Qatar-Shell GTL, Al Mirqab Tower, Doha), conducted in partnership with an ecological survey of vegetation and wildlife (S. B. El Din 2009 Ecology survey report of vegetation and wildlife for Qatar Shell proposed biodiversity offset investment at Al Reem Biosphere Reserve. Submitted to Qatar-Shell GTL, Al Mirqab Tower, Doha.), to inform discussions between the Ministry of the Environment and the gas and oil industry in Qatar over proposals to invest in a biodiversity offset programme in the Al Reem Biosphere Reserve, to compensate for the environmental damage for which the industry is responsible elsewhere.

The two surveys comprised brief two page questionnaires administered to a sample of individuals/households to collect data on social and economic arrangements, and to gauge local knowledge and opinions of Al Reem MAB Reserve (see Additional file 1, Appendix I for questionnaires). We administered some questionnaires directly during visits to the Al Reem region with the assistance of a team of student interviewers. Although this is the ideal way to conduct such a survey using trained interviewers, the problems that we encountered in finding and interviewing some members of the population prompted us to resort to the distribution of some questionnaires through schools, for respondents to complete and return. Seventy-five respondents completed the questionnaires. The survey data were entered onto Excel spreadsheets for analysis, using simple descriptive statistics.

The project survey team included both male and female students working in parallel during fieldwork, in order to access both men and women equally in the Al Reem region (in Qatar, where people subscribe to conservative Sunni Islamic tradition, it is not possible for male or female only research teams to access members of the opposite sex). The informal discussions were with men only, which may have introduced a certain unavoidable and unknown gender bias into some of the data.

One of us (AAS) is a Qatari of Bedouin background who, although belonging to a tribe (Al Marra) not represented in the Reem region, has some contacts there, as did some of the students assisting us. We visited many of the villages in the region and some of the stock camps to talk with people. The informal interviews took the form of semi-structured discussions. They often occurred in men's meeting houses (*majlis*) and sometimes in tents, frequently when several persons were present socialising over tea and coffee. Sometimes our stays involved sharing a meal. We allowed conversations to flow naturally, interjecting questions on issues of interest to us at intervals, particularly when the conversations veered off onto tangential issues.

These qualitative data were recorded and analyzed following standard ethnographic procedures [54-57], particularly those that follow social science triangulation procedures of the grounded-theory approach (that is, check the consistency or veracity of what persons tell us by going over the same issues with others and using different approaches), which places emphasis on providing evidence to support arguments [58-61]. Research into local environmental knowledge and ethnoscience is well established, encompassing such topics as ethnobotany, ethnozoology, cultural construction of the environment etc., and features a range of methods [62-65] that we intend to deploy in further research in the region. Some of this work includes enquiries into what conservation comprises in other cultural contexts [66,67], to which this work contributes from an Arab perspective.

## Humans and land in the Al Reem Reserve

**Figure 2:**
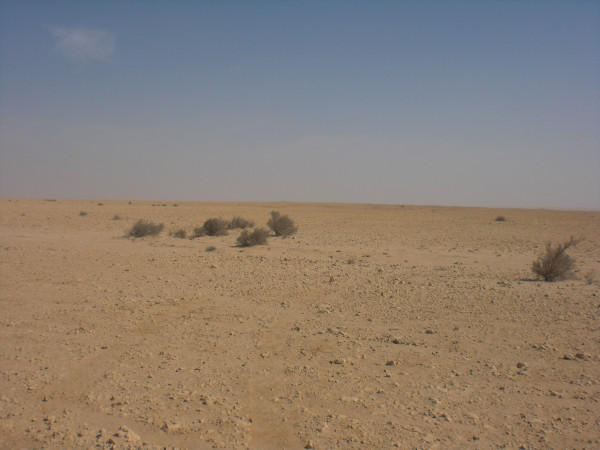
**A view across the Al Reem desert**.

The Al Reem Reserve comprises a fragile desert environment that experiences a harsh climate (Figure 2), where unsound land use can lead to serious degradation of natural resources. It comprises subtropical desert with hot humid summers and short semi-dry winters.

The changes that have occurred in land use and settlement during the second half of the 20^th ^century intimate the extent of the shift in people's relationship with their environment. Contemporary land access arrangements are a mix of the old and new - that is tribal customary practice combined with state bureaucratic regulation [68,69]. As noted by others elsewhere in Arabia, such as Gardner and Finan page 71, endnote 10 [70]: "Land tenure is a very complex issue on the Arabian Peninsula. Contemporary legal and bureaucratic structures overlay tenure principles drawn from Islamic law and customary practice. The combination of these forces active in a particular region at a particular time is, frequently, difficult to perceive."

**Figure 3:**
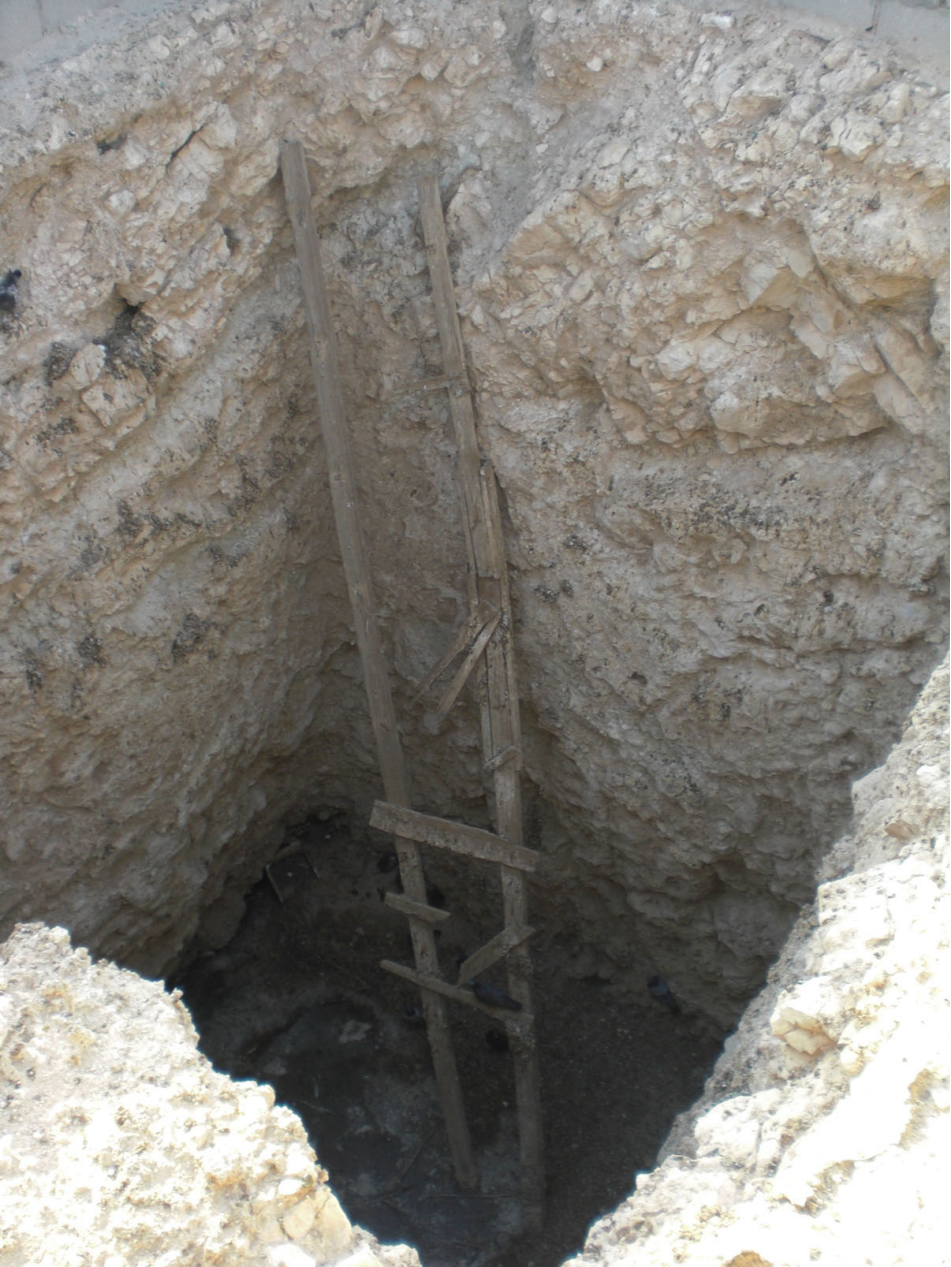
**A dry well, adjacent to Umm Al Qahab village**.

Tribal organisation has long structured socio-political arrangements in the region, and continues to inform it [see [71-75]]. The system basically comprises an arrangement of agnatically defined nesting groups of increasing size from the family up to the clan and tribe [76]. Descent defined groups are identified with certain *dirah *'territories' (see [68]), albeit they conceive of this identification flexibly. Such groups do not think of their territories as bounded areas to which they have exclusive rights of access [77]. According to Cole, page 33 [71], "Access to pastures blessed by rainfall ... [is an] open matter in which the rights of first come, first-served prevail", and Lancaster, page 123 [73], says that people "never felt that they owned the grass or water ... grass and rain come from God and is free to all". Livestock owners were consequently free to pasture their stock in any region where adequate pasture occurred regardless of tribal affiliation, pages 482-83 [53]. So if one region received good rainfall and had plentiful pasture, Bedouin came from far away to graze animals there. It was rights to water wells (*biyr*) that anchored tribes and clans to geographical locales [71] and pages 33-36 [75]. According to people they are centuries old (Figure 3); in the past, they sometimes fought over access. They were the location of summer tent encampments, called *ed *'place adjacent to well' or *bidiy *'water trough'.

A consequence of this rangeland access system was not only that people spread the risk of poor pasture in any season and shared the benefits of abundant pasture, but also that they moved over large areas. There are reports of pastoralists moving not only across large parts of Saudi Arabia but also as far as Kuwait, Jordan and Iraq. While there is no call for people to move stock such large distances today with tanker water and imported fodder, some continue to move stock between Qatar and Saudi Arabia. The manner in which they transport stock, often in flat bed trucks, signifies the different place of these animals in society today, where previously they carried humans and their possessions. The reason given for this movement is differences in Government fodder subsidies. While those who register their animals with the Qatari authorities^1 ^are eligible for a 50% subsidy, fodder is cheaper in Saudi Arabia. While some Qatari nationals may have stock in Saudi and have camps there, the Saudis do not apparently come in opposite direction and use kin/clan connections to herd stock in Qatar. The differences in fodder prices are probably a factor, and there are also legal obstacles regarding registration of livestock and campsites.

The sedentary attitudes of Qatari animal herders may have historical roots, their movements not being as extensive as those living in other parts of Arabia because the Qatar peninsula enjoyed better rainfall previously and families did not necessarily have to move large distances to find pasture. Also, many of them were not solely dependent on nomadic pastoralism for their livelihoods but participated in other aspects of the local economy; some of those living near to the Gulf coast worked in the pearling industry in the summer months, moving on with their herds for the rest of the year. Nomadism has ceased in recent decades. Control of wells has transformed into the right to build permanent dwellings and establish villages (*kariya*) adjacent to them [78]. The significance of these locales for water is reflected in some of their names.^2^

There are several small settlements dotted around the Reserve, many located off the highway that bounds it (see Figure 1). According to Ministry of the Environment staff, the numbers of households in the villages of the Al Reem region are shown in Table 1: Number of Households (Permanent settlements).

**Table 1:** Number of Households (Permanent settlements)

*Map no*	*Settlement name*	*No house-holds*	*Map no*	*Settlement name*	*No house-holds*
1	Zekreet	19	8	Al Busayyir	8
2	Khouzan	6	9	Abu Sidrahh	5
3	Umm Al Qahab	14	10	Al Suwaihliya	8
4	Al Refaiq	15	12	Umm Kheesah	5
5	Al Ejlah	1	14	Al Ghuwairiya	~65
6	Jemailiya	~75	15	Ain Al Numaan	6
7	Al Hawafer	1	17	Umm Al Edham	3
*At the following places, migrant workers only:*					
11	Umm Al Maa		20	Mazraat Umm Al Sheikh Abd Al Aziz	
13	Al Sakhbariya		21	Al-Uwiya	
16	Ras Eshairig		22	Al-Su'lukiya	
18	Al Sulaimi		23	Musa'b	
19	Umm Juwaiad		24	Aba Al Zubar	
					

**Figure 4:**
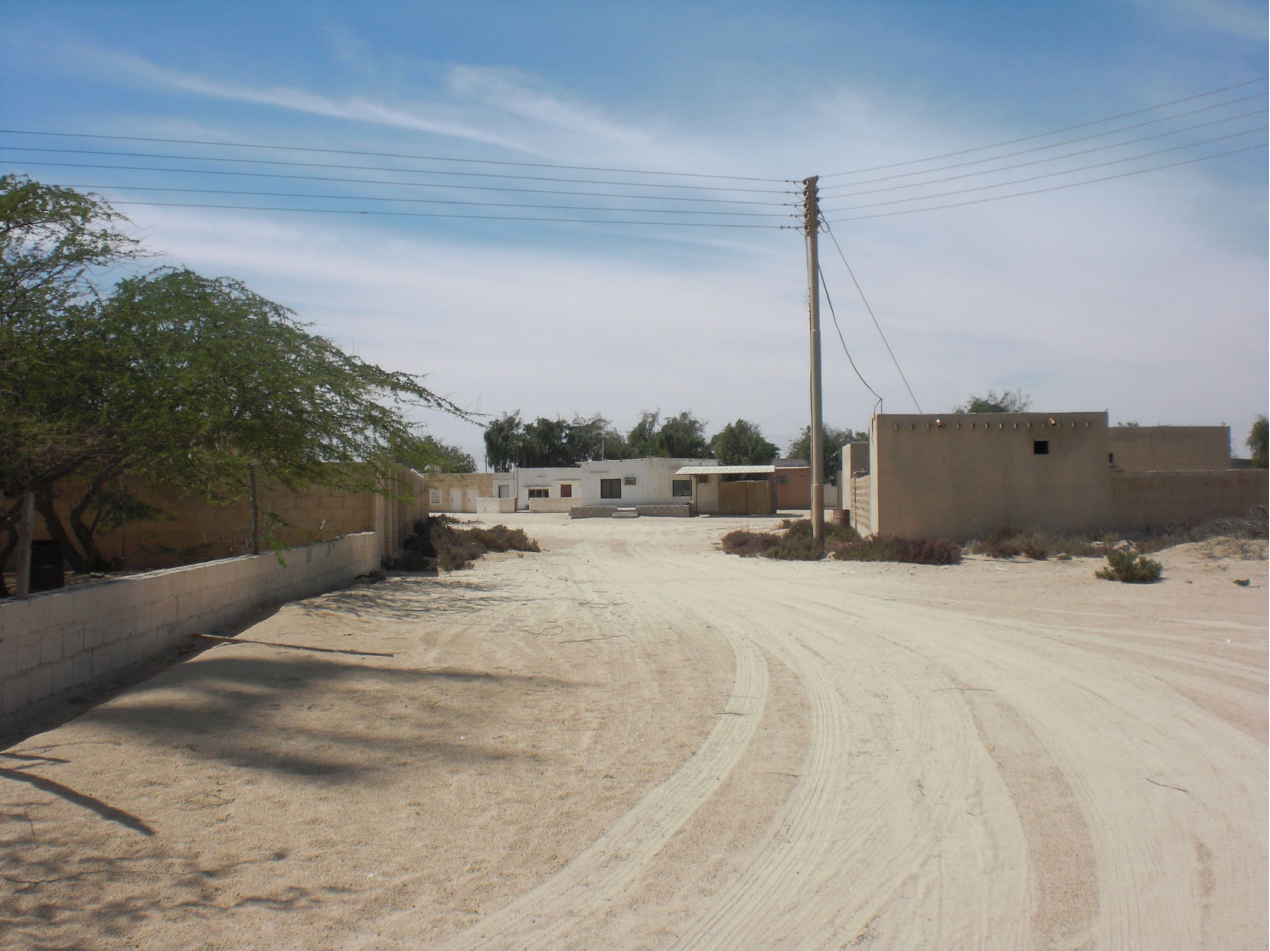
**The main thoroughfare of Zekreet village**.

**Figure 5:**
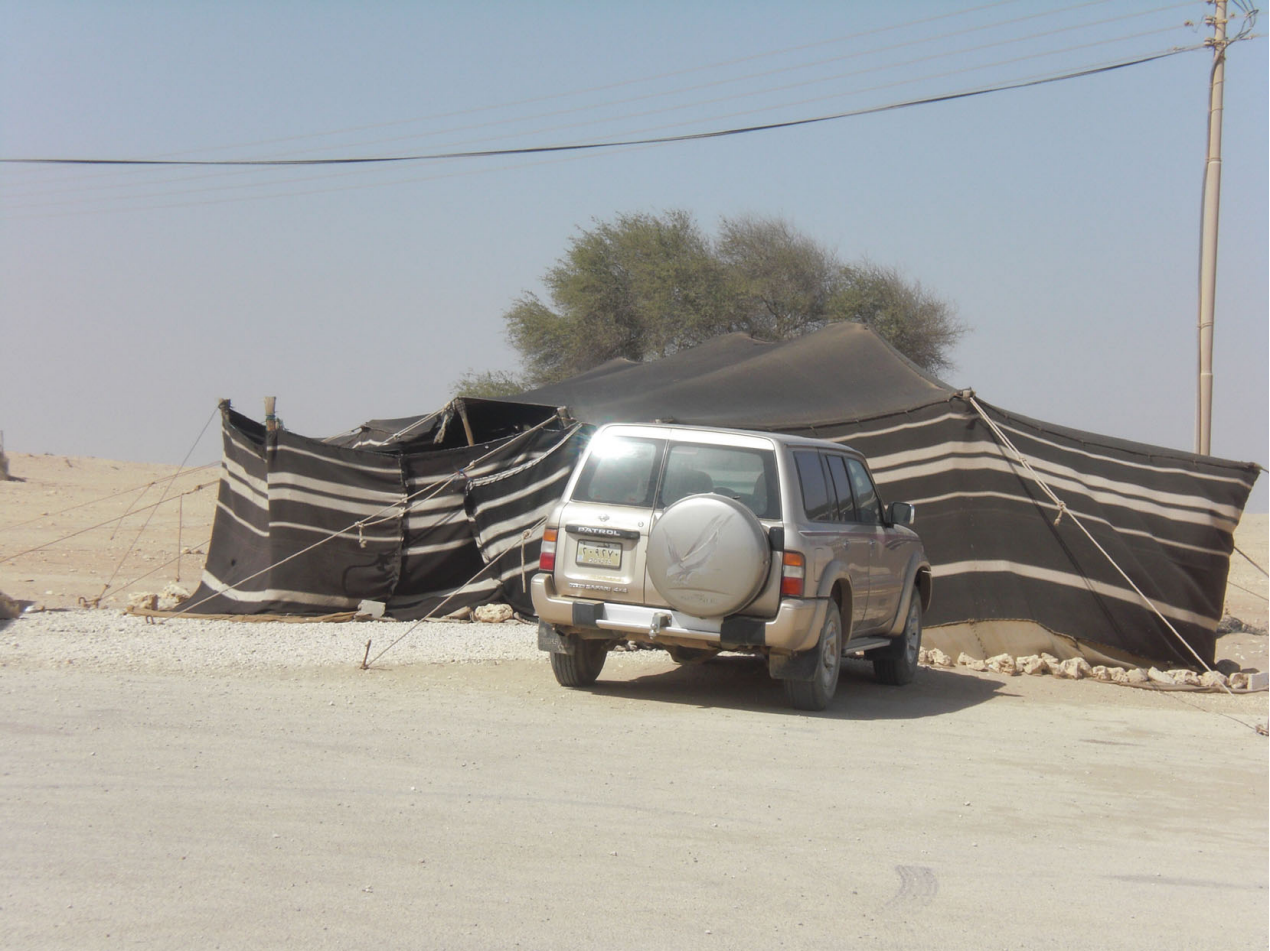
**Tent and vehicle on outskirts of Refaiq village**.

**Figure 6:**
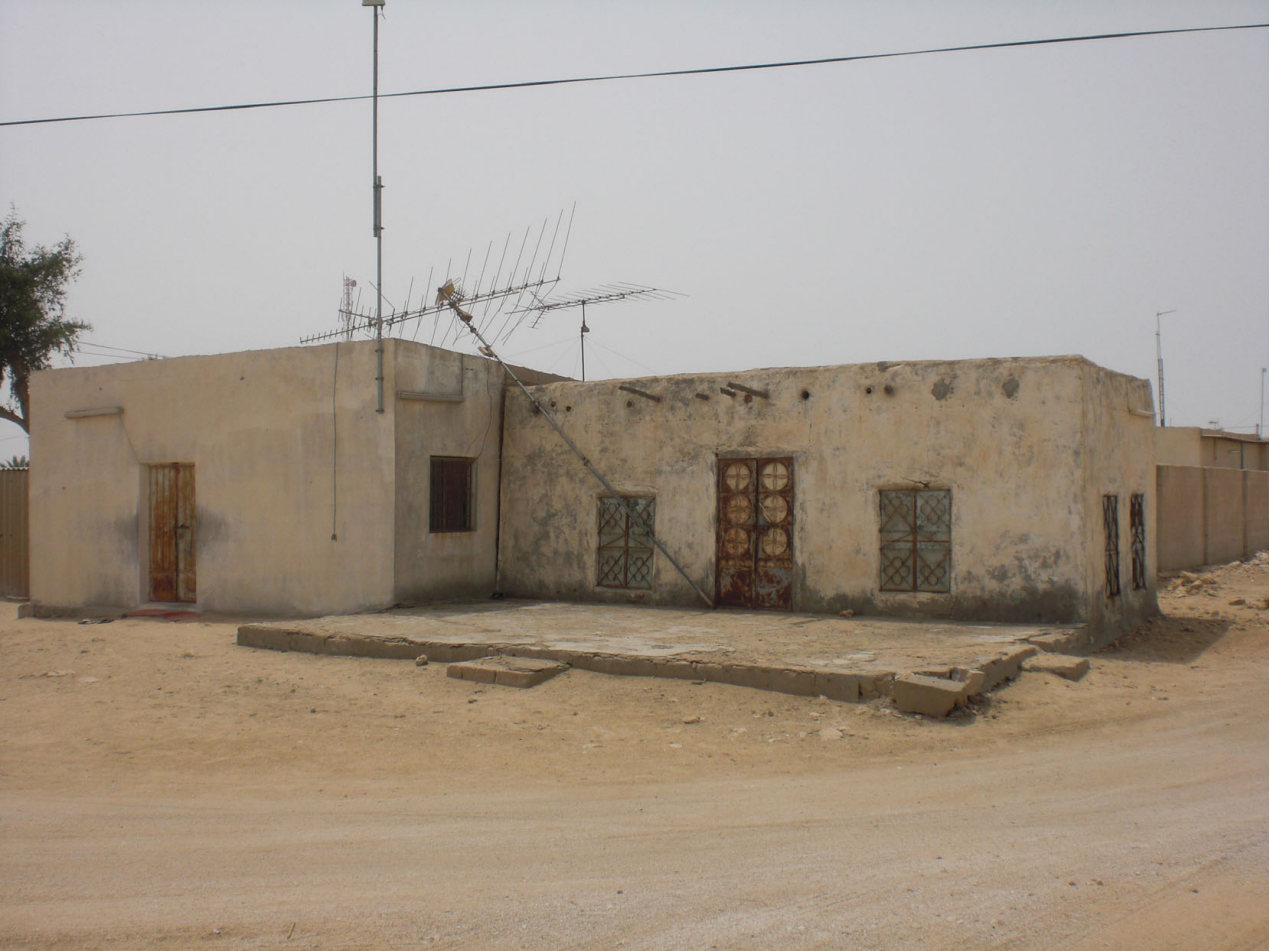
**A house at Umm Al Qahab village**.

**Figure 7:**
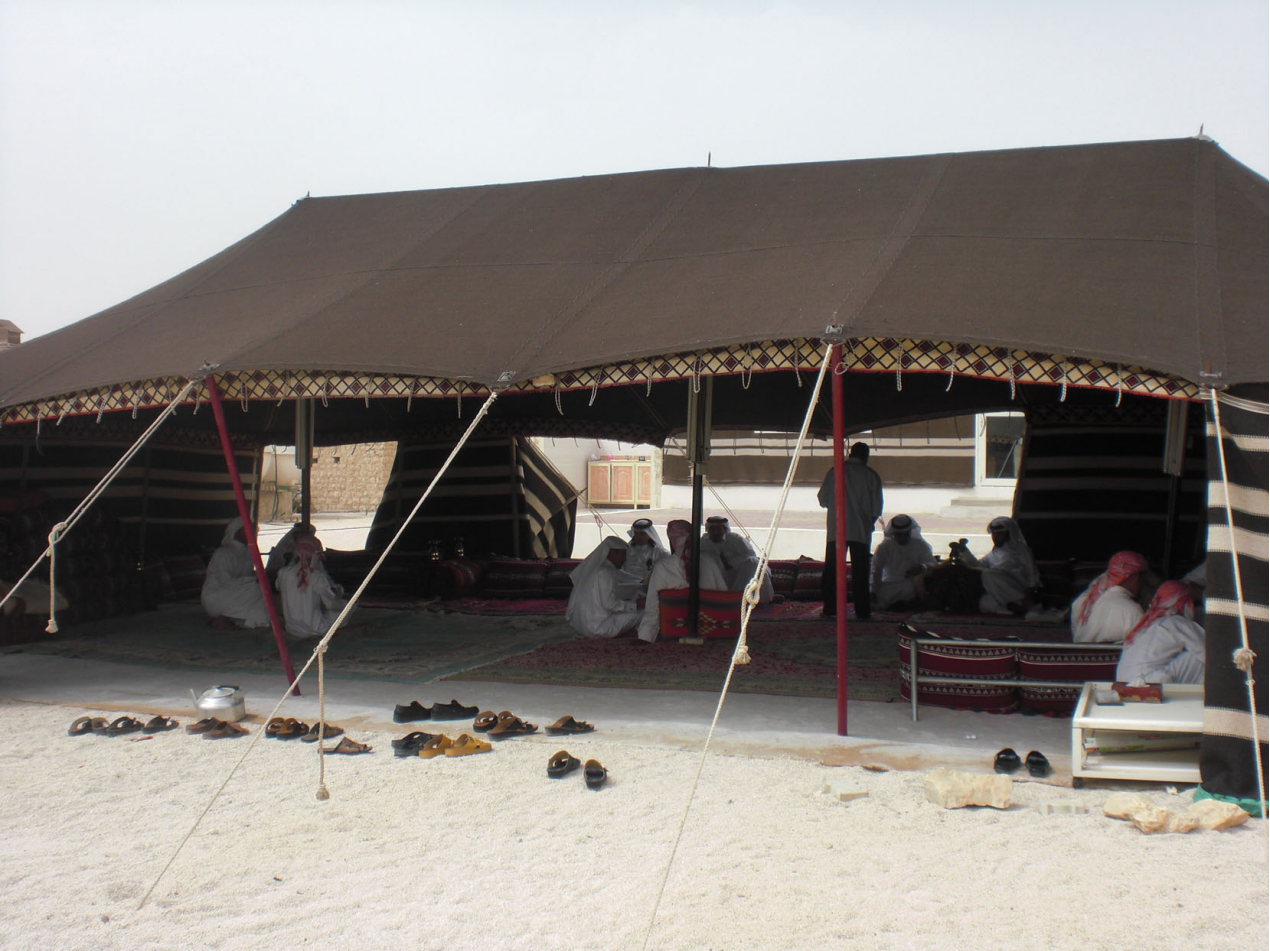
**View inside day tent at Zekreet village**.

Those living in villages currently talk about their fathers/grandfathers settling down and building houses with the development of the Dukhan oilfield, probably in the early 1950s, where many of them found work as labourers. Other clan relatives working for the oil companies joined the village founders. The village populations have grown, until today they largely comprise *bideda '*extended families' of kin. Sometimes people refer to villages as *al hezara *'immigrant [locales]', changed places where families now live 'newly' (like immigrants) all the time. They comprise modest family houses, often around a small *masjid *'mosque' and *majlis *'men's day room' (Figures 4, 5, 6 and 7). Both within the village and on the outskirts are various enclosures and buildings for animals. There are small areas under cultivation, including some *tamar *'date palm' groves. Although traditional wells are currently dry, some of the water used for irrigation comes from local sources, diesel pumps tapping into ground water via tube wells. Drinking water, together with further water used for irrigation, is brought to villages in water tankers from municipal deep wells.^3 ^This threatens longer term, even irreversible degradation, by both depleting aquifers faster than rains can replenish them, water supplies eventually diminishing, and increasing soil salinity, until the land can no longer support crops.^4^

**Figure 8:**
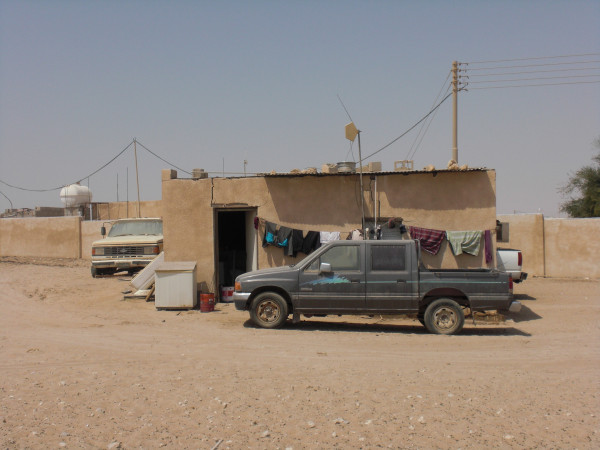
**A migrant workers' house at Refaiq village**.

While the majority of residents lived permanently in these settlements until the 1980s, many have since moved to Doha for employment and schools, becoming weekend visitors. Those who continue to live there permanently are largely farmers with livestock, although some have other occupations, such as school teachers (who may engage in some farming too). Those residing in the region fall into two groups according to nationality, either Qatari or other. Households employ many migrants, largely Asians, as labourers and domestic servants (Figure 8). The large numbers of such labourers, comprising something like eighty percent of the country's population, are an aspect of the dramatic social changes that have occurred with exploitation of petroleum reserves [79].

**Figure 9:**
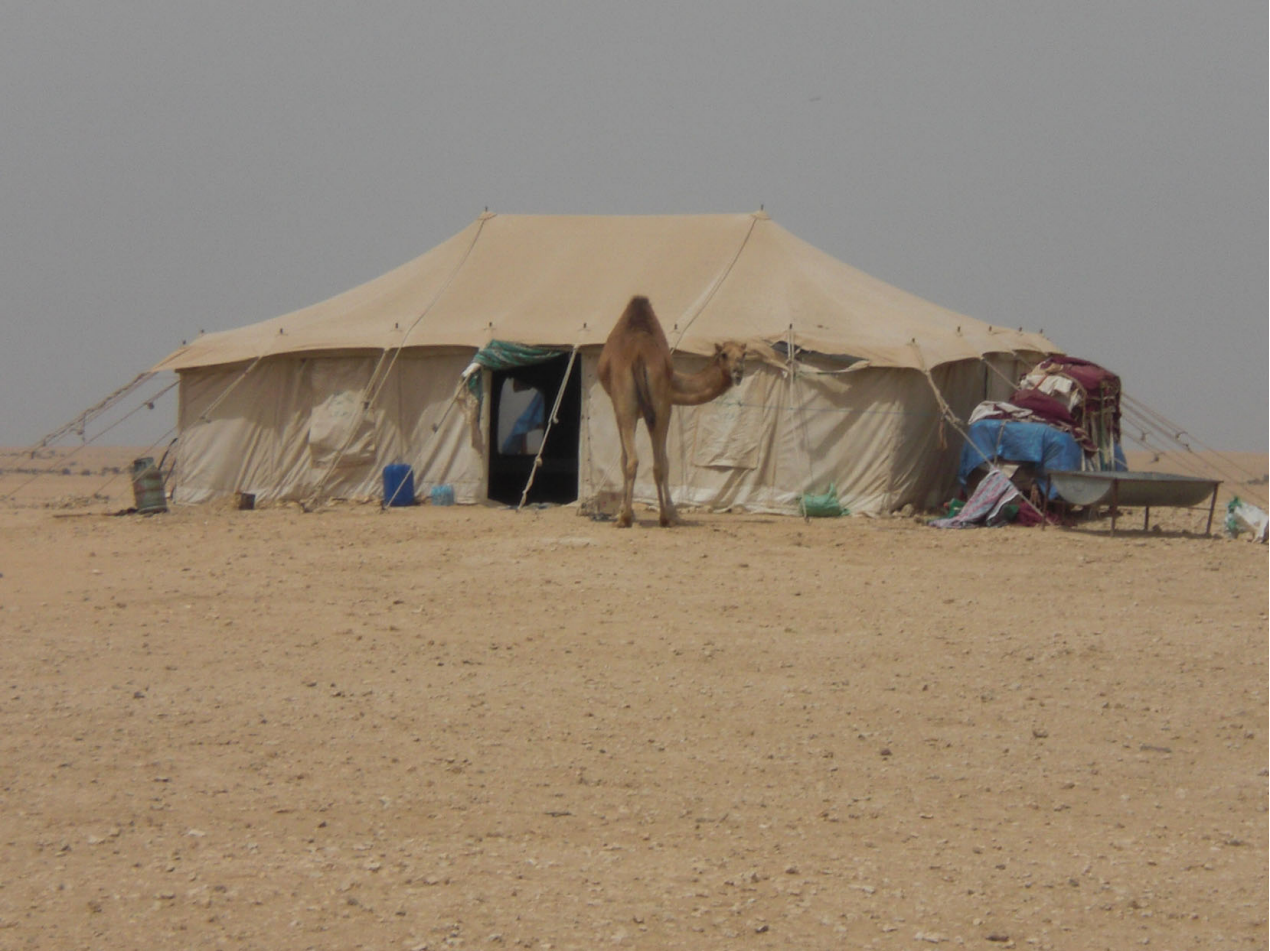
**Camel herding camp at Al'jla**.

**Figure 10:**
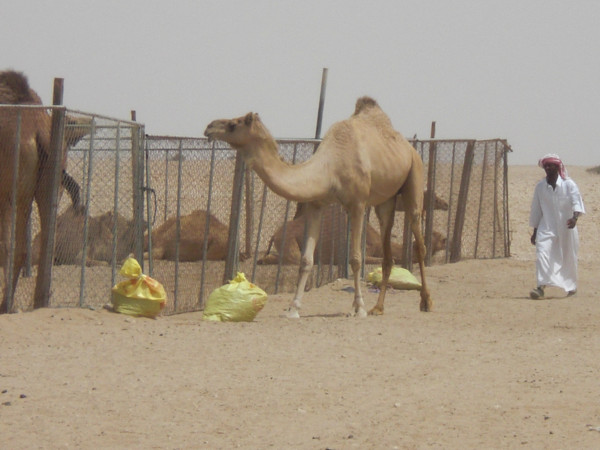
**Rounding up camels into pen at Al'jla camp**.

**Figure 11:**
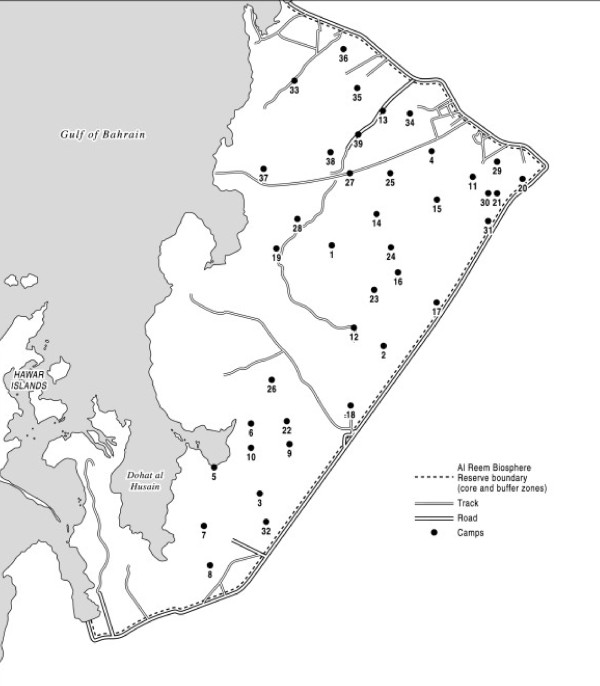
**Camp locations**.

Temporary herding camps (*mukaiyem*) also occur in the region (Figure 9). While only Qataris can apply for a licence to establish such camps,^5 ^those living at them are overwhelmingly (if not exclusively) non-Qataris such as Sudanese, Bengalis and Nepalis, who herd the stock kept there (Figure 10). There are on average between four and eight migrant workers residing at such camps. Similarly, elsewhere in Arabia: "The everyday herding of almost all the livestock on the Saudi Arabian range is left to hired shepherds, usually expatriates" page 121 [44]. According to data supplied by the Ministry of Municipal and Agriculture Affairs, there are thirty-nine camps in the Al Reem Reserve (see Figure 11)

When selecting a camp place, people say that they look for somewhere there are no others, either encampments nearby or homesteads. Camps where livestock are herded are invariably situated inland because of the danger of animals wandering onto the *sabka *'salt flats' adjacent to the sea, falling through the saline crust and becoming stuck and dying. Some stock-camps are established by villagers in the desert away from their settlements; others belong to persons from outside the region. The majority of Qatari men with residence rights in the Al Reem region keep some animals. According to staff in the Jemailiya office of the Ministry of Environment, there were sixty-four village dwelling permit holders in 2009 who herd stock in the region, who may or may not maintain stock-camps elsewhere from the village (Table 2: Temporary Camps^6^) (Figure 11: Camp locations)^7^

**Table 2:** Temporary Camps

*Location name & Map no*.	*Location name & Map no*.	*Location name & Map no*.
**Jemailiya Municipality:**		
1. Abu Suf	12. Qatna	23. Sini' Alghadriyat
2. Al-Humailat	13. Qura Abu Raghed	24. Sulaimiyat Bu'ina
3. Al'jla	14. Qura Alqaraf	25. Um Alkhuraq
4. Al-Khashina	15. Rowdat Azza*	26. Um Al-Zabd*
5. Alnafayed	16. Rowdat Khalifa	27. Um Juwaid*
6. Al-Qamirya*	17. Shamal Al-Gazlaniya	28. Um Khayeesa
7. Barbiyat	18. Shamal Al-Jamailiya	29. Um Qarn
8. Baseeteenat	19. Shamal Alsuwaihla	30. Wady Al-Ja'da
9. Fushakh	20. Sharq Al Ghuwairiya	31. Wasee'
10. Janoub Bu Ghamara	21. Sharq Wady Al-Ji'da	32. Widyan Alsahm
11. Juda	22. Shukeek	
**Madinat Al-Shamal Municipality:**		
33. Alfahadat	36. Janoob Lushai'	39. Shamal Al-Nu'man
34. Idgheesheeyat	37. Maroob	
35. Janoob Abwab	38. Nu'man*	
		

Five have no licensed stock and are recreational camps only (marked *). The Ministry has Qura Abu Raghed camp incorrectly listed in Jemailiya Municipality.

The State bureaucracy features a system of permits that controls the establishment of stock herding camps and hunting activity. It is necessary to obtain a permit issued by the Ministry of the Environment to establish a camp and also to register animals kept there; rules that apply to all rural Qatar. You can only have one camp at any time in all Qatar but can move from one location to another, having secured a permit for the new place. There are two broad classes of stock-camp permit holder: *mazarah*, persons who own 'own earth', that is have permanent houses in the region, and *azba*, non-residents who have a permit to establish a temporary camp.

The Ministry staff check that the animals are appropriately registered with the Department of Animal Resources at the General Department for Agriculture Research and Development. This is undertaken for health reasons - to monitor stock movements and know the whereabouts and numbers of animals in the event of disease outbreak - an aspect of the considerable changes in herding practices. The system of regulation, which is subject to local political manipulation, is now allowing persons without a pastoralist heritage to herd animals by legitimating access to territory that previously would not have been possible without necessary tribal connections. The blurring of customary practice by state intervention parallels the changes that are occurring in herding arrangements.

## Demography and degradation

The traditional strategy of open competition between herders for pasture is not only compromised today by bureaucratic restriction of access to rangeland and sedentary lifestyle but also population growth. There is no census of those living in the Al Reem Reserve region. The aforementioned UNESCO MAB Nomination File (page 14) estimates the permanent population from the 2004 National Census (according to Zones within Municipalities and human settlement distributions) to be 400-500 persons in the core area and ~8,000 in the buffer zone (three-quarters male). According to Ministry of Environment briefing notes, the total population comprises 11,160 persons. While these statistics are on the high side, and presumably include the populations of Jemailiya and Al Ghuwairiya towns, they give a density of population way beyond what the region supported previously.

**Figure 12:**
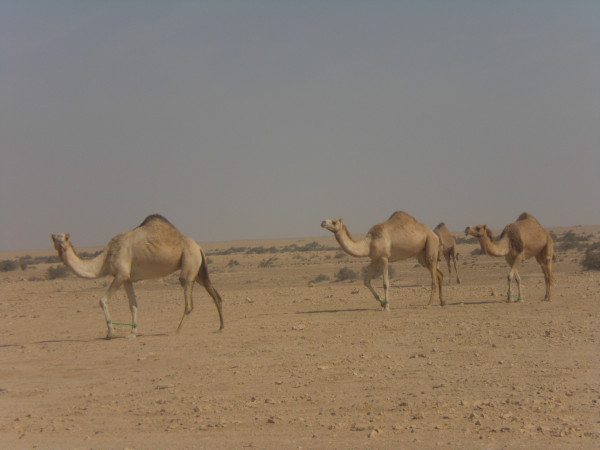
**Free ranging camels in Al Reem region**.

**Figure 13:**
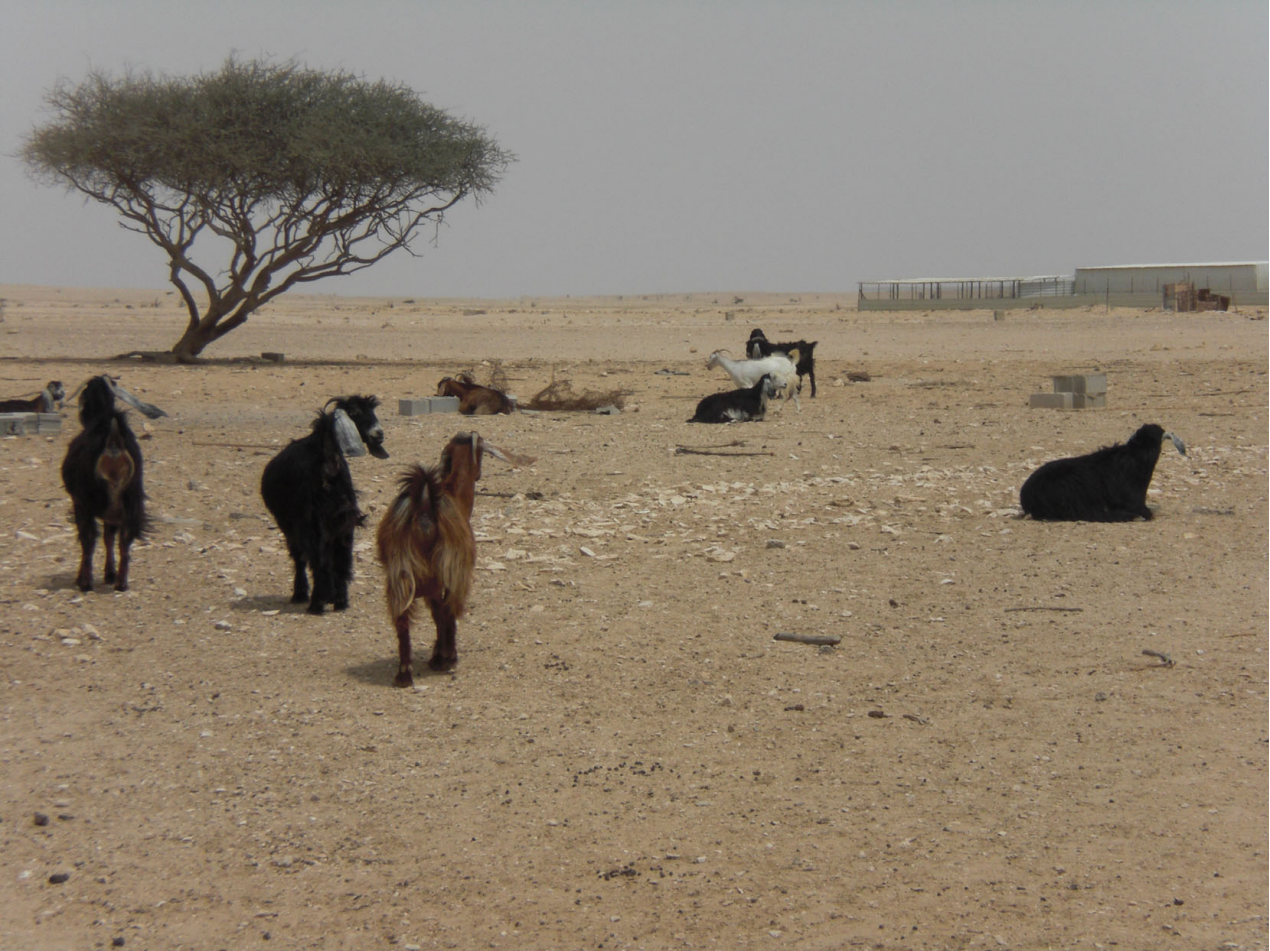
**Free ranging goats on outskirts of Umm Al Qahab village**.

It is the large numbers of animals that these people herd that are thought responsible for environmental degradation (Figures 12 &13), being way beyond the region's natural carrying capacity, pages 483-85 [53]. While calculation of carrying capacity presents difficulties, available stock figures and contemporary herding practices suggest that it is exceeded by a large margin (Table 3: Village animal numbers (*mazarah *rights) in Al Reem region^8 ^and Table 4: Stock-camp animal numbers (*azba *rights) in Jemailiya & Madinat Al Shamal Municipality regions of the Al Reem Reserve^9^)

**Table 3:** Village animal numbers (*mazarah *rights) in Al Reem region

	Camels	Cattle	Sheep	Goats
No. villages with	12	12	16	16
No. homesteads with	32	29	52	41
Total animals	728	272	4806	2653
Mean per homestead	22.8	9.4	92.4	64.7
Range by homesteads	1-99	1-90	2-402	3-220
Standard deviation	22.9	16.6	90.3	47.3

[Total nos. villages & homesteads owning stock = 16 & 56] Data in this and the following table (of March 2009) kindly supplied by the Department of Animal Resources (Ministry of Municipal and Agriculture Affairs).

**Table 4:** Stock-camp animal numbers (*azba *rights) in Jemailiya & Madinat Al Shamal Municipality regions of the Al Reem Reserve

	Camels	Cattle	Sheep	Goats
No. camps with	22	7	25	21
Total animals	803	57	2528	1059
Mean per camp	36.5	8.1	101.1	50.4
Range between camps	1-102	3-15	2-285	2-129
Standard deviation	27.8	4.2	75.4	36.7

[Total no. camps owning stock = 39] Five locations had two separate stock licenses in different names and treated as separate camps.

These data are indicative only. We might assume that one snapshot in time gives a representative indication of stocking levels, but monitoring animal numbers is difficult. For example, while there is the 50% fodder subsidy incentive for owners to register their animals, they do not necessarily do so, but may transfer plastic registration ear-tags from slaughtered animals to others.^10 ^Also, stock numbers can fluctuate, possibly widely according to comparative data in Ministry of the Environment briefing notes.^11 ^The variation in animal statistics probably reflects to some extent the fact that stock currently herded in Al Reem region is not confined there but may be moved considerable distances elsewhere, which further confounds attempts to calculate herd levels commensurate with conservation.

But such stock movements are minimal compared to previously. Today herders are constrained not only by national borders and a system of government imposed permits but also by internal borders and highways. The Al Reem region is tiny compared to the area the Bedouin previously roamed over [71,68,75]; land degradation seems unavoidable with so many animals kept in such a relatively confined space. While sustainable herding seems a forlorn hope at current stocking levels, there is evidence that resources may previously have sometimes been inadequate too. Periodic fighting and raiding of stock - celebrated today in poetry at large social events such as weddings - is possibly evidence that resources were insufficient on occasions, leading to violent confrontations over rangeland and water sources [80]; Chatty, page 11 [43], refers to "frequent skirmishes ... as tribes struggled to lay claim to the most fertile parts of the semi-arid and arid lands".

**Figure 14:**
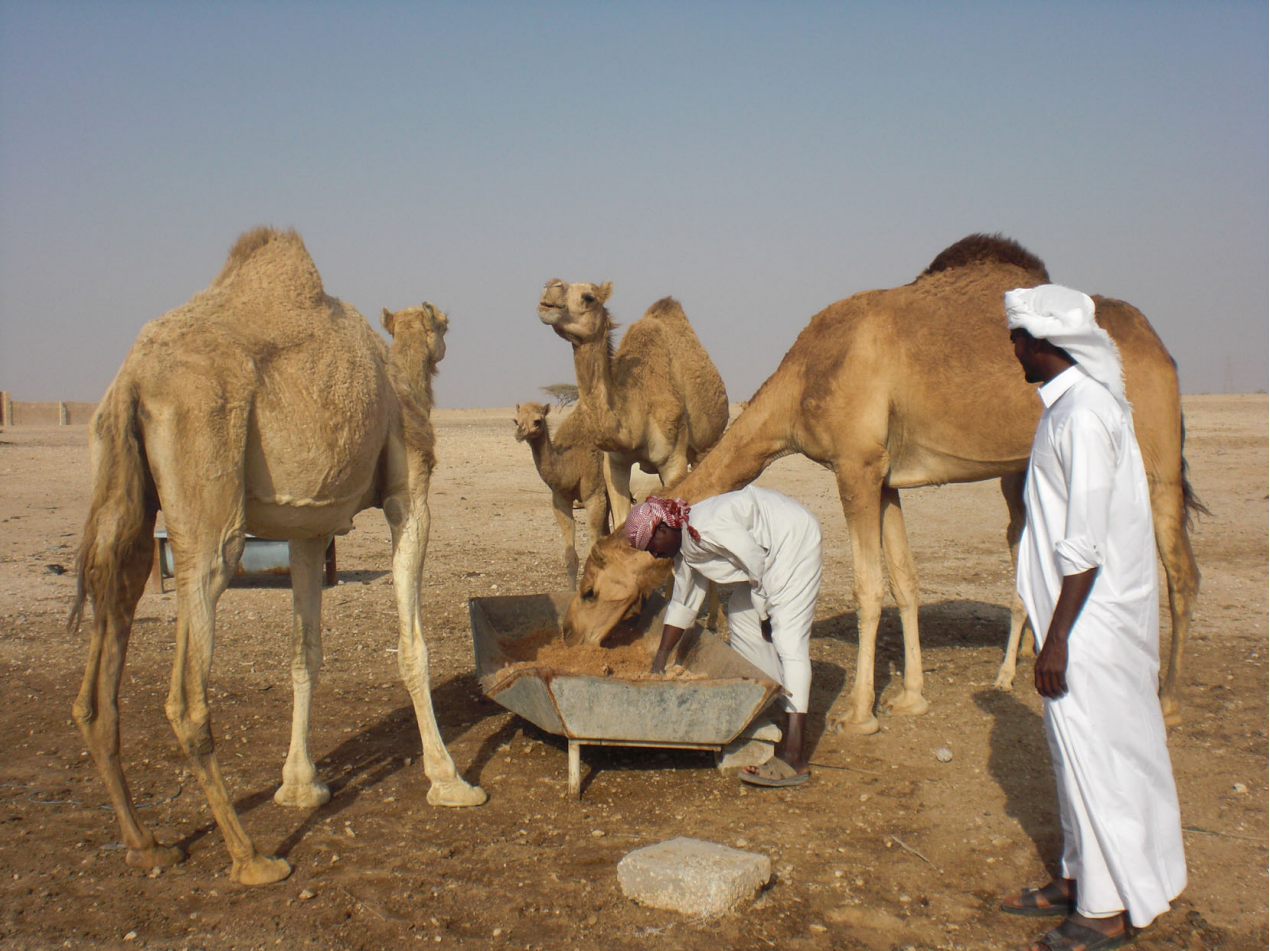
**Feeding camels oatmeal ration**.

**Figure 15:**
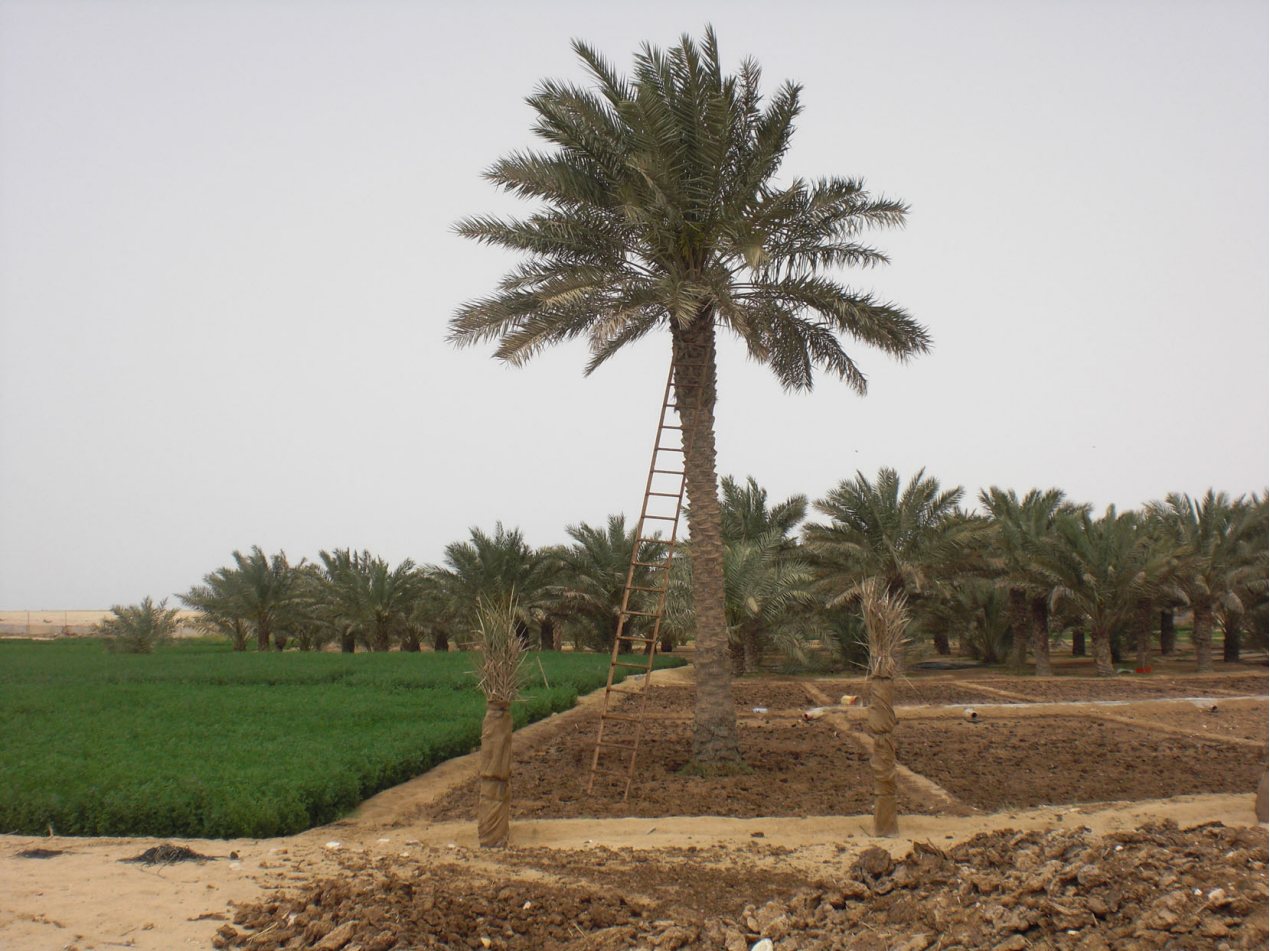
**Alfalfa and date cultivation at Umm Al Qahab village**.

When stock exceeded rangeland resources previously, the ecological safety valve was animal deaths, reducing numbers to levels supportable on available resources; there are historical reports of people losing many animals during droughts. Today's coping mechanism, when carrying capacity limits are exceeded beyond anything imaginable previously, rests on the import of fodder and water. Animals depend on a ration of imported feed, the sheep and goats largely on *alef *'alfalfa' (*Medicago *spp.) and the camels on *shuwar *'oatmeal' mixed with water Figure 14); other fodder includes barley, *rodus*, *jibt*, dry bread and dates. The alfalfa cultivated on irrigated plots of land in some settlements (Figure 15), particularly in the northern Al Reem region,^12 ^is nowhere near enough to feed current numbers of animals.

## The logistics and logic of animal herding today

**Figure 16:**
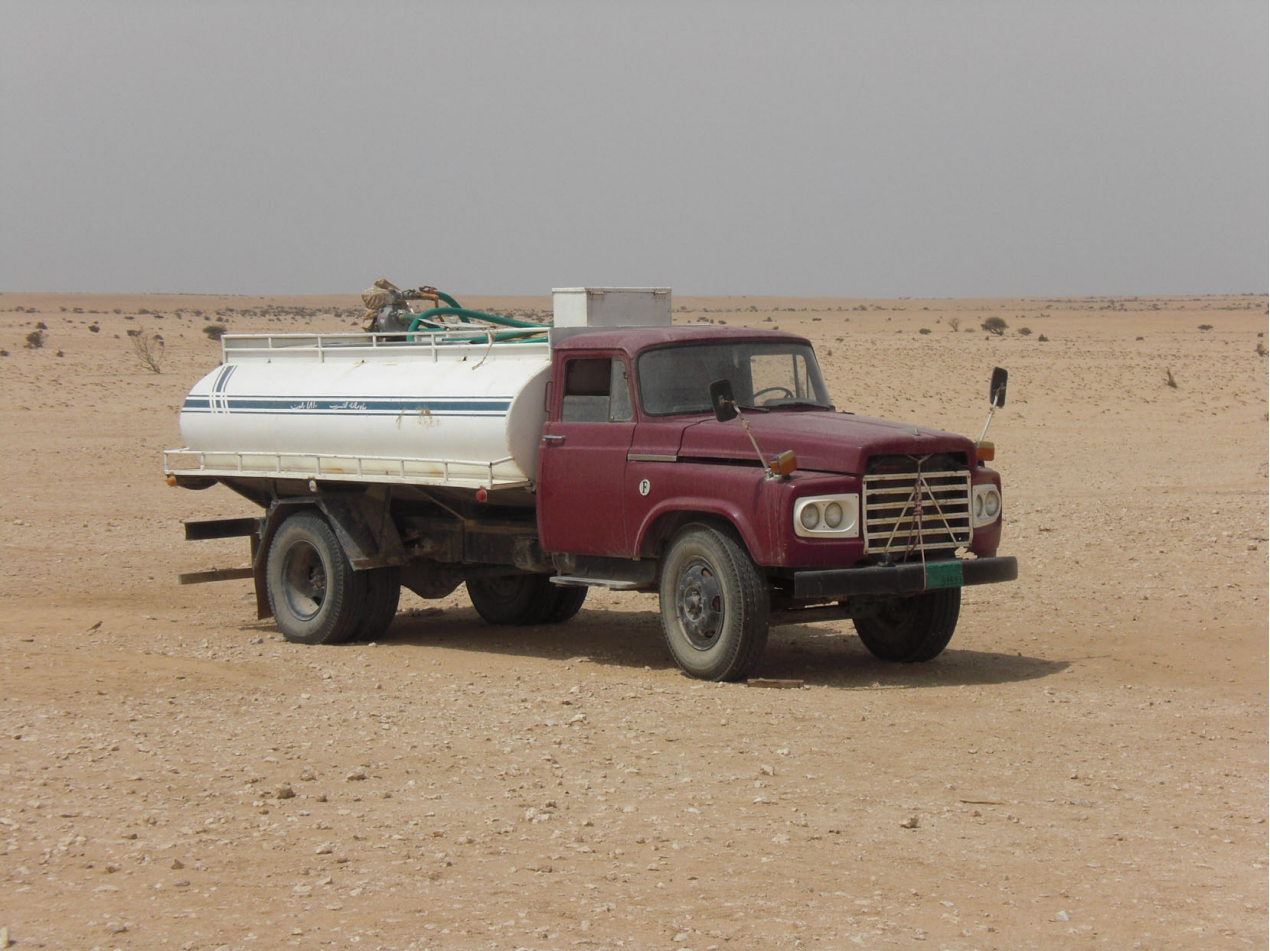
**A water bowser parked at stock camp**.

The environmental costs of herding are partly exported elsewhere through the import of fodder, much of it from overseas, and trucking of water (Figure 16). This compromises assessment of the costs, the local region not being a closed ecological system regarding herding activities. The locally unsustainable character of contemporary arrangements - way beyond available local resources - is not apparently an issue currently, with potentially significant conservation implications. There is no competition over available grazing, to trigger any ecological readjustment. The current regime depends on oil and gas revenues that underpin Qatari employment and subsidise people's herding activities - such that they can afford to purchase the large volumes of imported fodder necessary to keeping so many animals and truck water to otherwise barren places. Participation in the global marketplace has changed their relationship with their environment. A question from a conservation viewpoint is the extent to which we are witnessing a state sponsored so-called 'tragedy of the commons' [52] with government subsidised fodder and water supplies and permit controls promoting environmentally damaging changes in traditional communal land management strategies.

The large numbers of animals relative to local water supplies and pasture resources not only reflect the dramatic changes that have occurred in animal husbandry in recent decades [46] and pages 111-34 [44] but also the changing place of animals in Qatari life, the very reasons for keeping stock. While some families continue to earn a proportion of their living through raising sheep and goats,^13 ^few depend on camel herding to any extent. Nonetheless some people continue to keep large numbers of camels. Although some camel owners and their families in Al Reem villages, and migrant workers at stock-camps, consume some camel milk and meat,^14 ^many now keep camels not primarily for food but as status symbols, for demonstrating social standing and wealth.

**Figure 17:**
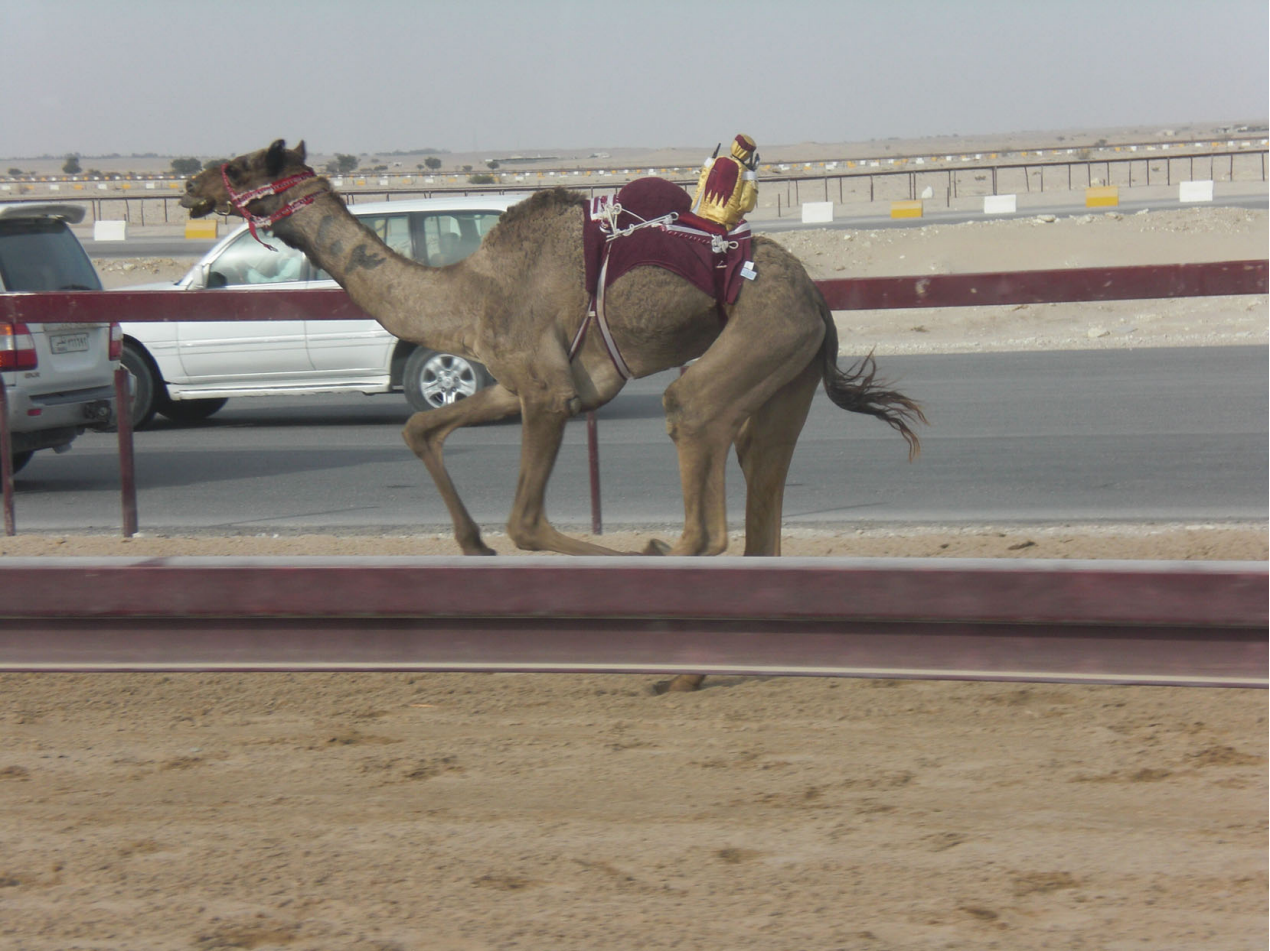
**Racing camel with robotic jockey, Al Shahaniya racetrack**.

While camels traditionally featured as wealth in Bedouin communities, they were previously linked directly to subsistence requirements - a successful man could support a large family from his herd, a mark of social standing and renown. Today, camel ownership is still the mark of success for some, animals serving as stores of surplus wealth, rather like gold jewellery. It is an intriguing transformation of custom with globalisation [81,42,82], camels serving today as investments of income deriving from the country's vast hydrocarbon reserves that permit the above unsustainable arrangements. In a review of the changes that have occurred during the three decades that he has known the Al Murrah tribe, Cole, page 380 [83], talks about purebred camels taking "on a new value in a prestige economy that was emerging". Camels sell for tens, even hundreds of thousands of Riyals (some exceed a million Riyals). There are shows at which animals judged best (by size, body shape, coat colour and deportment) can win their owners large sums of prize money (a million or more Riyals). Other highly valued animals are juveniles that can run fast, which are trained as racing camels [84]; again winners earning their owners large sums (Figure 17).^15 ^The parallel with race horses is apposite, in which the rich also invest large sums.

The keeping of camels is a feature of Gulf Arab cultural identity in a globalising world [85], page 103 [73], page 380 [83]; for former desert dwellers it is something that marks them out, while ensuring some continuity with their past.^16 ^We should not expect economic logic to apply when something achieves such iconic status. When discussing fluctuations in the price of fodder, for instance, informants said that it does not influence the number of animals they keep; so price manipulation may not prove particularly effective in trying to protect the environment from perceived over-grazing - even if politically acceptable. There are significant implications regarding reserve management where animal owners invest disproportionately in their herds for status reasons rather than to earn their livelihoods.

While participatory approaches to development are thought the best way to ensure incorporation of local knowledge and practises into projects, we should not romanticise these latter, as not all local understanding and aims inform activities that necessarily respect biodiversity, particularly in times of rapid change. We have to ask if ownership of camels, goats and sheep is too important an indicator of social status for people to contemplate modifying current practices and restricting the size of their herds. So long as they can import fodder and truck in water, is the state of the rangeland immaterial? In other words, current economic arrangements allow expression of the cultural estimation of stock ownership without regard to environmental costs, whereas not so long ago such attitudes would have cost nomadic herders their livelihoods by degrading the pastures on which they and their animals depended. Albeit, as Cole comments ominously, "The new pastoralism is fragile, a luxury" page 391 [83].

## Back to prohibition?

It appears that a focus on bio-cultural diversity to promote and manage conservation may breakdown with rapid social change, the cultural element transforming to such an extent that human activities become environmentally destructive. The restricted movement of people and stock today questions the applicability of local knowledge to environmental management when this knowledge and associated practices depended on more extensive movement of people and stock [24]. As one man in Zekreet observed:

"There is no benefit of having *hima *grazing [former fallow pasture management system] in Qatar, because Qatar is a desert country that only has a small amount of land."

The assumptions of co-management are compromised and we have the re-emergence of the 'Yellowstone model' of national parks that prohibit or heavily control human activities to protect the environment. We see this in UNESCO's plans for the Al Reem Biosphere Reserve, which feature aforementioned core conservation zones [where people will be excluded and nature strictly protected from human activities] and a buffer zone [where protection measures will be less stringent].^17 ^But this prospect leads to number of contradictions.

We return to the issue of what sort of environment we seek to conserve. The 'Yellowstone model' aims for pristine nature untouched by human activity, whereas we know that the environment we see in the Al Reem region today is partly due to generations of human activity, so excluding people will presumably result in a changed, albeit eventually 'natural' environment (so far as possible anyway with global hydrological and atmospheric systems). But we have the prospect of a changed - and in ecological terms degraded - environment anyway, if current human activities continue, which have apparently gone haywire with respect to the local ecology, no longer sustainable and damaging the ecosystem. Either way the environment may differ from previously, as people are unlikely to revert to their previous nomadic livelihood strategy.

A Yellowstone-type park seems an improbable prospect politically, even though the Government owns 90% of the region (UNESCO 2007 'Al-Reem Reserve: UNESCO MAB Biosphere Reserve Nomination File', submitted to The Supreme Council for the Environment and Natural Reserves, State of Qatar page 32), previously held under the tribal common property regime. It would require a large extension of government authority beyond current arrangements, which as described, involve the Ministry of Environment overseeing the licensing of campsites and the registration of animals. In theory the current licensing system gives the government the authority to regulate animal numbers, but there is scant evidence of any attempt to do so in Al Reem region. To go further than current arrangements seems politically unlikely, as it would require intruding into people's lives more than probably acceptable. Current reserve management and the attitude of rangers reflect political reality, albeit to the frustration of international agencies, which see the declaration of a reserve without any apparent action to make it a reality. The frustration felt at UNESCO is evident in a recent *Gulf Times *article [86], which refers to desertification, biodiversity loss, water crisis, pollution and climate change, commenting that while some Gulf states have "established biosphere reserves to reconcile development and nature conservation. This needs to be promoted".

While the current Al Reem situation suggests the prioritizing of environmental and biodiversity protection, extending to the imposition of rules to regulate access to, and use of the region's resources, this may not politically be feasible. The Biosphere core and buffer zones drawn up by UNESCO extend to 1,190 square kilometres and the transition zone (which falls outside the Al Reem Reserve area as declared protected by Emiri Decree) takes the area to 2,024 square kilometres or nearly 18% of the country (UNESCO 2007 'Al-Reem Reserve: UNESCO MAB Biosphere Reserve Nomination File', submitted to The Supreme Council for the Environment and Natural Reserves, State of Qatar page 4). While Qatar is an absolute monarchy, where the Emir is both head of state and government and directly accountable to no one, there is a tradition of consultation and rule by consensus, symbolised in every citizen having the right to appeal to the Emir personally. He and the government he appoints are obliged, in the interests of political stability, to consider the opinions of leading civil and religious notables, such as tribal Sheiks who represent the views of their fellow tribesmen. It is unlikely that popular opinion would support measures to make Al Reem a protected area where access and use of resources are strictly controlled or that the government could enforce such without serious social unrest, as it would amount to excluding the population from a large part of the country.

## The participatory park

The 'participation model' is the only viable option. The issue is how to facilitate participation that supports conservation. Local Qataris are already participating in Reserve activities. Many rangers are Al Reem residents who herd animals and hunt there. Daily management is under their control via the Ministry of Environment in Doha. But the local populace is not otherwise involved and understanding of issues is patchy, as the current apathy of rangers indicates. When asked about his views of restrictions on hunting, for instance, one answered:

"I have four hunting dogs they are large, like this [indicating with his hand] and I go hunting often in season. I have two falcons too and I do not think that it would be good to stop me or my brothers hunting like our fathers."

Some agreement over conservation issues is necessary before people will countenance any protection measures that regulate access to and use of their region's resources. The results of the survey conducted to assess people's awareness of and attitudes towards Al Reem Reserve and conservation issues give grounds for optimism. According to these, 64% of respondents knew that Al-Reem was designated a MAB Reserve and 69% thought that this was a good thing. Peoples' understanding of conservation and what it implies suggests less consensus. While a large proportion - 69% of respondents - claimed to know what conservation is, only 7% could provide a definition, such as, "It is preserving the environment."^18 ^When asked whether they thought conservation was necessary, only 45% of respondents answered yes.

Some spoke out against the declaration of their region as a Biosphere Reserve. Why have it, they asked, when there was so little rain and so scarcely any vegetation to protect and little pasture for animals anyway, however managed? As one man commented:

"No point in having a reserve in Qatar because there is no rain in the desert areas; also this region was established for habitation before it was a reserve; we do not want anything to impose on Qatari culture."

They also see the Government as not straight dealing, pointing out for example that the army conducts exercises in the region, damaging plants and greatly disturbing the wildlife, how can this square with having declared a Reserve there? And conservation attempts by the Ministry of Environment have furthered scepticism. Attempts to reintroduce *reem *gazelle into the region have been mismanaged, resulting in the death of many animals. According to Ministry of Environment briefing notes, in April 2007 there were one-hundred-and-twenty gazelle [released?] in the Al Reem region. An elderly man at Zekreet described how the Ministry had employed him on a small stipend to look out for the gazelle (to provide water and fodder as necessary) but suddenly stopped doing so. "Who," he asked angrily, "is looking after the gazelle now?" "No one," he replied, "so in hard times like now they are dying." Such experiences understandably make residents somewhat cynical of talk about a Reserve and conservation.

Grassroots political views generally complain that the Government does not know what problems those living in the region face. Many of the issues encountered in the course of the survey are similar to those reported elsewhere in the Gulf region where there are similar plans to promote biodiversity conservation (cf. D. Chatty n.d. Adapting to Biodiversity Conservation: The Mobile Pastoral Harasiis Tribe of Oman. Unpub. Typescript). There is understandably widespread concern about the extent to which the declaration of the region a Reserve will affect lives, such as imposing new restrictions on the herding of animals or right to hunt. On the other hand, there is considerable support detectable for investment that might protect or even improve the natural environment - which it is widely agreed is showing signs of degradation, albeit local views of the causes may differ from outside authorities.

## Local villains or victims?

During discussions, some persons argued that they and their forbearers have always been good custodians of the environment. They were affronted at the suggestion that their activities, notably grazing of animals, were responsible for the apparent environmental degradation seen in the Al Reem region. For example, the first page of the UNESCO MAB Nomination File refers to "desert gravel plain ecosystems ... partially degraded through overgrazing," and elaborates further later "the greater-than-carrying capacity density of grazing animals ... has reduced natural grazing material to extremely sparse densities ... This trend needs to be reduced through zonation and limitations on grazing animals" (UNESCO 2007 'Al-Reem Reserve: UNESCO MAB Biosphere Reserve Nomination File', submitted to The Supreme Council for the Environment and Natural Reserves, State of Qatar page 30). There is a risk of stereotyping [25], imposing the foregoing tragedy of the commons view [see [52]] on a land use system, which as indicated, is intricate and needs to be understood in all its complexity. For instance, Chatty, page 12 [43], points to an "academic critique ... [of] international and national land use paradigms which have sought to blame the Bedouin for what was widely regarded as man-made land degradation and desertification ... questioning government claims of widespread desertification and range degradation due to Bedouin overgrazing and other pastoral activities."

**Figure 18:**
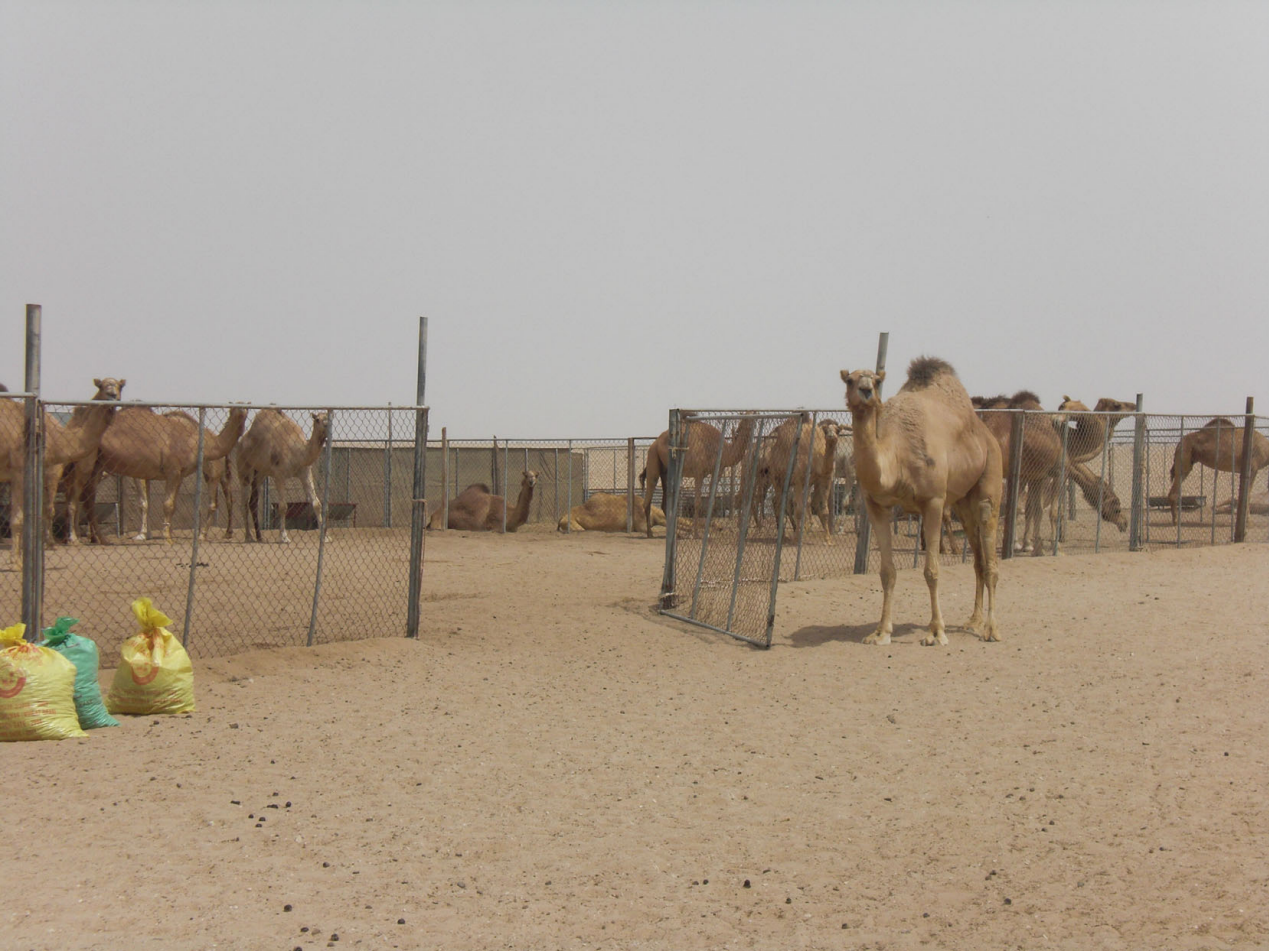
**Camels in pen at Al'jla camp**.

**Figure 19:**
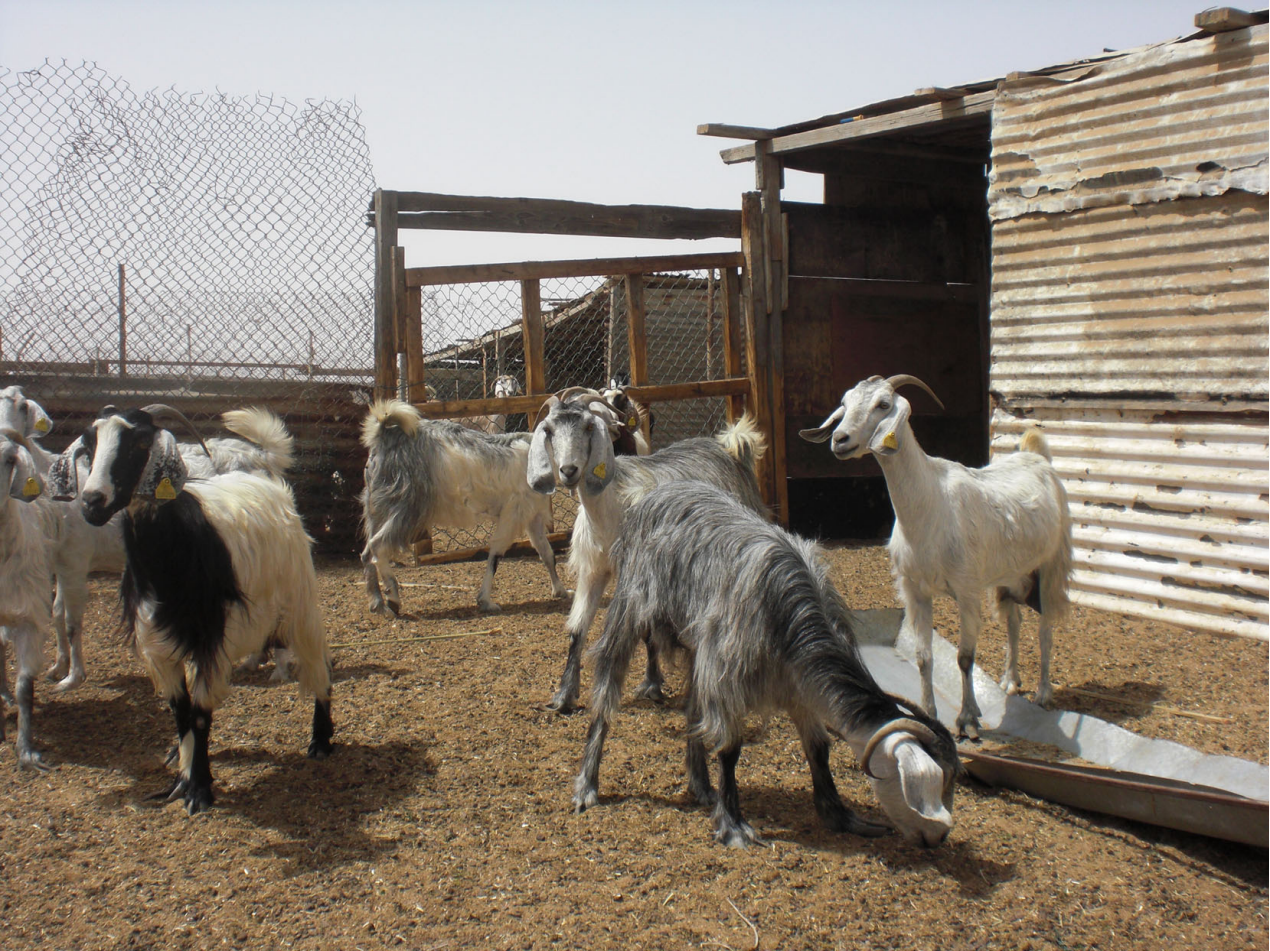
**Goats in pen**.

**Figure 20:**
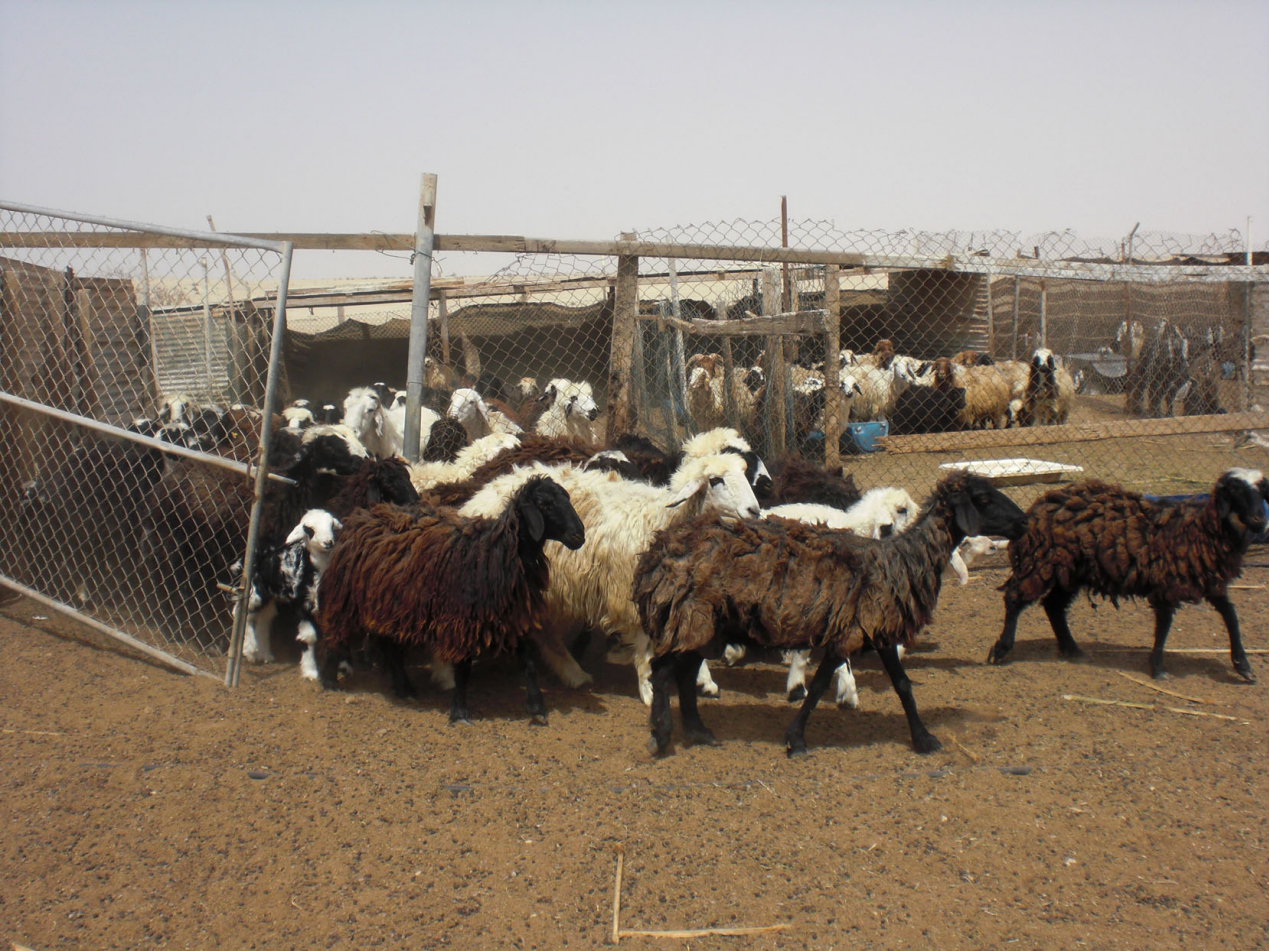
**Sheep in pen**.

Local people point out that they have herded stock in the region for generations without undue destruction of the vegetation [87]. And a recent survey of vegetation in the Al Reem region supports such claims, concluding that "human activities do not have a significant impact on either species richness or vegetation cover" page 49 [88]. The owners of stock, whether kept in villages or camps, manage their animals closely. Many animals - camels, sheep and goats - are kept in pens during the daytime, probably because of the poor grazing available locally (Figures 18, 19 &20). Camels, sometimes hobbled, may be allowed to roam unattended during the day, returning to village or camp water source of an evening. Goats and sheep, on the other hand, need shepherds in attendance if allowed to browse in surrounding country (similarly in neighbouring Saudi regions, page 485 [53]). They are herded near village or camp, whereas camels may wander further off. While some camels roam free and graze what they find in the desert, and some goats wander free in villages, they are few compared to total numbers, which suggests that it may be inappropriate to blame the poor condition of vegetation on over-grazing. This is certainly the view of local people. They point out that having a shepherd control flocks means that the animals do not roam freely and graze vegetation destructively, particularly goats that are notorious for heavily browsing plants. Camels, on the other hand, they point out are more fastidious feeders and after nibbling some foliage from a plant move on to the next one.

**Figure 21:**
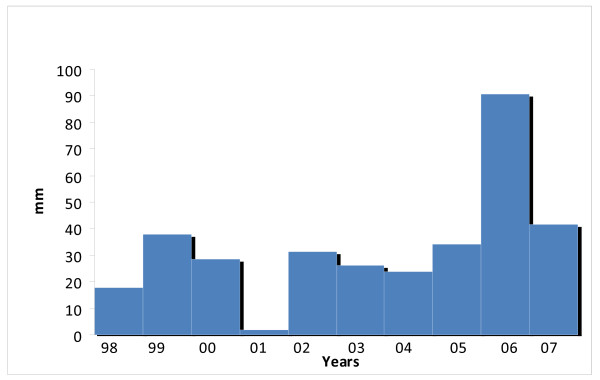
**Rainfall [total annual]**.

**Figure 22:**
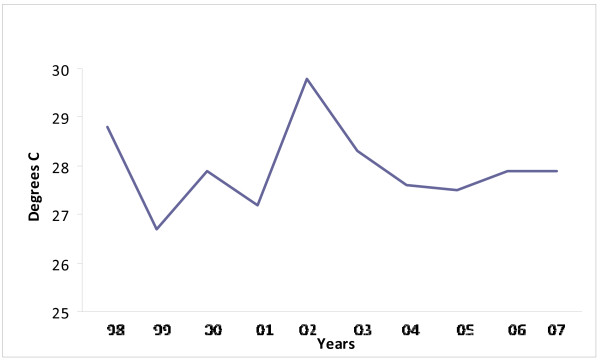
**Temperature [mean annual]**.

When asked what they thought was responsible for the current poor condition of vegetation in the region, if it is not overgrazing or poor animal management, people said that it was due to lack of sufficient rainfall for several years past. "There is no rain in the desert areas nowadays, it is less than in our fathers' time before", as a Refaiq man commented. Some persons went on to attribute the deficiency in rainfall and the region's consequent desiccation to the will of Allah, punishing people for their sins. As someone commented: "The greed of wealthy families is sinful, they are not sharing fairly as the Koran tells us, the money that is coming from Qatar's oil and gas." According to others, recent droughts, possibly attributable to climate change, have affected the quality of range in the region [70]; see [89] for recent account of Mongolian herders' views on subject). While current climate change predications suggest decreased and erratic rainfall for the Gulf region, climate data from Dukhan (the nearest meteorological station to the Al Reem region) do not support the view that the region is experiencing either declining rainfall or increasing temperatures (Figures 21 &22 - also see Table 5^19^). The extent to which environmental change and degradation is due to climate change is debatable and demands further research, which is expectable as the issue globally is currently subject to dispute. Whatever, local ideas about environmental degradation need to be taken seriously regarding Reserve management.

**Table 5:** Meteorological data

Rainfall (mm) at Dukhan [Lat : 25 25 N Long : 50 46 E; Elevation : 30 m]													
						
Year\Month	Jan	Feb	Mar	Apr	May	Jun	Jul	Aug	Sep	Oct	Nov	Dec	Total

1998	17.9	0	0	0	0	0	0	0	0	0	0	0	17.9
1999	1	37	0	0	0	0	0	0	0	0	0	0	38
2000	4.8	0	0	0	0	0	0	0	0	0	1.6	22.2	28.6
2001	1	0	0.7	0	0	0	0	0	0	0	0	0	1.7
2002	31.5	0	0	0	0	0	0	0	0	0	0	0	31.5
2003	0	0	0	6.2	0	0	0	0	0	0	0	20.2	26.4
2004	0	0	0	3	0	0	0	0	0	0	0	21	24
2005	19.4	7.8	7.1	0	0	0	0	0	0	0	0	0	34.3
2006	0	7.4	3.3	11.7	0	0	0	0	0	0	10.4	57.7	90.5
2007	4.8	31.5	2.5	2.8	0	0	0	0	0	0	0	0	41.6

**Mean Dry Bulb Temperature (Deg. C) at Dukhan**													
Year\Month	Jan	Feb	Mar	Apr	May	Jun	Jul	Aug	Sep	Oct	Nov	Dec	Total

1998	18.2	21.6	24.6	27	29.7	36.6	36.6	37.9	33.3		22.3		28.8
1999	16.4	18.7	20.8	26.5	30.1	33.9	34.6	35.2	32.9	29.3	24.5	17.6	26.7
2000	17.2	17	20.5	28.4	32.3	34.2	37.7	37.3	33.5	30.6	25	21.2	27.9
2001	18.5	19.7	25	30.1	35.8	34.9	33.6	35.1	32	26.5	19.1	16.3	27.2
2002	13.3	20.2	25.2	29.6	36.8	36.3	37.2	35	33	31.3			29.8
2003	17.2	19.8	22.5	28.2	33	35.6	35.8	36.8	33.6	31	24.9	20.7	28.3
2004	20.7	20.1	22.7	26.1	31.2	33.8	36.6	34.8	32.5	29.5	25.1	18.1	27.6
2005	16.2	17.3	22.3	28	31.8	34.2	35.5	36.1	33	29.2	25.2	20.9	27.5
2006	18.3	20.1	22.8	27.8	33	35.6	36	35.9	33.2	30.8	24.8	16.3	27.9
2007			21.6	26.7	32	33.8	34.8	34.4	31.2	27	22.1	15.4	27.9

## Local knowledge in conservation

In order that the local population can participate meaningfully in planning and implementing of conservation management measures it is indubitably necessary that it has the necessary knowledge. It seems that many Qatari may be unaware of the consequences of their actions in a world greatly changed from that of their grandparents, not that they are indifferent to their peninsula becoming a virtual moonscape dotted with urban sprawls. Understanding of what the designation of the Al Reem region a MAB Biosphere Reserve implies appears limited from the survey returns. For example, we should expect a large majority of people to know that they are living in a Biosphere Reserve and the implications for the region, whereas only 33% claim to know anything about planned management interventions to improve the state of natural resources. In this regard, more awareness of conservation is an educational issue (UNESCO 2007 'Al-Reem Reserve: UNESCO MAB Biosphere Reserve Nomination File', submitted to The Supreme Council for the Environment and Natural Reserves, State of Qatar pages 27-28), informing people about the issues so that they appreciate the urgency of taking action and the majority support it, so making a participatory park a reality. This is to reiterate UNESCO's point about the need to promote "public awareness activities aimed at varying sectors of society" (UNESCO 2007 'Al-Reem Reserve: UNESCO MAB Biosphere Reserve Nomination File', submitted to The Supreme Council for the Environment and Natural Reserves, State of Qatar page 8). Any education policy could profitably draw on the extended family group structure of local communities to disseminate information widely via kin networks.

But education takes time - even generations to have an effect - while the environmental problems are urgent and demand conservation measures today [90]. There is also a danger that promoting education might be seen as advocating ethnocentric brainwashing to the Western conservation view. In this event, biodiversity conservation may become a Western imposition to be resisted, or at least the reasons given for advocating conservation in the Reem region, namely that interventions are necessary to counteract the actions of the local population responsible for degradation. Any education programme should respect what people think and know, and exercise cultural sensitivity in promoting awareness of, and discussion of environmental issues, so that the participatory park model becomes a reality and they may conclude for themselves what conservation action is necessary. Such promotion of locally informed discussion and avoidance of ethnocentric impositions, opens the prospect of building on cultural practices, beliefs and knowledge to further conservation efforts [91,30,92,49,22]; that is, co-opting Arab concepts & experience to the Reserve's conservation ends, so involving the local population more meaningfully in its management, for they may better appreciate its aims if clearly related to what they already know and believe [23,37]. This reiterates another point made in the UNESCO MAB Nomination File about the importance of local knowledge featuring in any future plans for the Reserve; where it talks, for example, about "the use of traditional ecological knowledge in research studies" (UNESCO 2007 'Al-Reem Reserve: UNESCO MAB Biosphere Reserve Nomination File', submitted to The Supreme Council for the Environment and Natural Reserves, State of Qatar page 9). It relates to the possibility of modifying and reinstating bio-cultural diversity assumptions.

The fact that humans have lived in the region for millennia in a way that has maintained natural resources sufficient to sustain their livelihoods from generation to generation indicates, as argued above, that they followed practices that promote biodiversity conservation [87], even if they had no such words or conscious ideas. In establishing a cultural ethic of valuing the environment, culturally appropriate approaches to conservation education might draw on references to nature in both ancient and modern Arabic literature, for deferring to the authority of Islamic teaching and heritage will further efforts to promote conservation [93,94]. This relates again to the theme of Arab cultural identity [95-97]. It accords with the aims of the *Qatar National Vision 2030 *(see foreword by Tamim bin Hamad Al-Thani) to build "a bridge between the present and the future ... [in a] prosperous country ... in which nature and man are in harmony" and traditional Arab cultural values "provide our moral and ethical compass" [6]. For as Mozah bint Nasser Al-Misnid expresses it, "We need to care for our natural environment for it was entrusted to us by God to use with responsibility and respect for the benefit of human kind. If we nurture our environment, it will nurture us" see page 30 [6].

A key question is the extent to which such environmental knowledge and practices are still extant, and then to ask how the Al Reem Reserve authorities might profitably employ them. For instance, is there a working memory of the traditional *hima *community based grazing arrangements of Bedouin pastoralism that left areas fallow for vegetation to regenerate [98,25], pages 32-34 [99], pages 787-88 [26], sufficient to further the objectives of biodiversity conservation? In order to answer this question it will be necessary to further understanding of the complex *hima *grazing system and ask how people might be persuaded to reinstate, if discontinued, or innovate on it to reverse rangeland degradation if, as many assume, overgrazing is a problem. When we raised the issue of the practice of *hima *rotation of grazing areas, some maintained it continues in modified form. As a man from Khouzan commented: "it is good because it preserves animal resources", while another thought that the imposition of such arrangements would be "bad because we will feel like we are imprisoned." Again, this is to mobilise people's sense of identity as herders of animals, through such practices, to further the objectives of biodiversity conservation, which is to turn round one of the perceived causes of environmental degradation to serve its protection. In addition to cultural and aesthetic considerations, there are straightforward practical ones too, such as using concerns for the welfare of animals to promote more conservation oriented behaviour and practices, by reducing overcrowding and attendant risks of disease and injury.

While Bedouin pastoralists may have followed practices that protect natural resources from overexploitation, current behaviour suggests that these may not equate with a Western-like awareness of the need for conservation. Otherwise, why do people, including reserve rangers mandated to promote conservation, evidence such apparent indifference to contemporary environmental damage? It is necessary to beware of over-esteeming local understandings, as shown by surveyed people's opinions of various proposed conservation measures. There are four such interventions under consideration, one of which is the re-establishment of the community based *hima *grazing system, 56% supported the idea as "Good to spread understanding about the importance of the Reserve", while others thought it no benefit, "As Qatar only has a small amount of land". Of the other proposed measures, 49% thought that the establishment of exclosures (small fenced areas to keep animals out and protect seed banks) would be good for the region as "It will protect local shrubs and trees", while others thought it "Bad because it will limit freedom of movement".^20 ^53% thought control of vehicular access a good thing "Because it will protect the environment", while others thought it bad "Because it will limit our freedom". 56% thought that a prohibition on hunting locally is a good idea "Because it will preserve wild animal numbers", while others opposed it "Because it is the tradition of our ancestors".^21 ^The overall level of agreement was 54% for all four combined.

The idea of bio-cultural diversity and possibility of co-opting local knowledge and practices to conservation goals may imply the need to conserve culture too, as well as nature [100]. But this is a contentious idea with a controversial history featuring salvage ethnography and attempts to save traditions. The restoration of previous practices that promoted sustainable resource use will not be successful unless culture bearers can see the point and want to do so. If turning the clock back to previous ecologically balanced lifeways or excluding people and imposing Yellowstone park style conservation are inappropriate-cum-inadequate, a viable way to proceed may be to promote a new sustainable accommodation between human population and environment.

This relates to attempts by well meaning authorities to introduce outside-brokered conservation-framed interventions, such as proposals to promote environmentally friendly tourism, particularly eco- and cultural tourism [101], and camel dairy farms [102].^22 ^But similar to the reinstatement of previous sustainable land use practices, the development of such new practices that meet conservation's aims are only likely to prove successful if they resonate with local people and their expectations [103]. In other words, they need to be developed collaboratively to meet with local approval and to take root. Any imposition will probably prove unsustainable. Regarding tourism, the omens from the survey are mixed. Residents seem fairly tolerant of tourists, in view of the numbers passing through some villages, such as Zekreet, especially at weekends. They said that they are happy to see them enjoying the beaches and sea, so long as they do nothing wrong, such as speeding through villages or leaving litter. But some expressed anger at seeing visitors, particularly joy-seeking youngsters, damaging the land, driving recklessly off tracks, damaging vegetation,^23 ^or leaving rubbish after camping for a night or two (a permit is not necessary for such short stays), which they have to clear up so that it does not harm grazing stock; animals have been known to die because they ingest refuse.^24^

## Conclusion

Biodiversity conservation raises complex issues and we should not anticipate straightforward solutions to the problems posed by bioreserves such as Al Reem [27]. Central to the success of such conservation initiatives, as experience elsewhere in the world shows, is meaningfully to involve the local people so that they support any measures, ensuring that they fit with their socio-cultural expectations and their interests as resource users [48,49]. This point is made by UNESCO several times, for example, when it refers in the 'Al-Reem Biosphere Reserve Nomination File' to involving "stakeholders, and local communities in particular, in planning and implementation of conservation and management measures" (2007:9). The challenge is making such participatory parks a reality and not only rhetoric, particularly where rapid change occurs, upsetting people's previously more sustainable arrangements.

It can be difficult for deskbound policy makers, researchers and planners to understand the local viewpoint and allow it expression. For instance, concerns for freedom of movement, mentioned several times to us, are a common issue among pastoralist populations and have long featured in their relations with farming neighbours and nation states that have sought to curtail their freedom. In other words, it is a deep rooted issue. It has wide implications pertaining to core cultural values about individual autonomy. It can be challenging to appreciate the significance of such values and accommodate to them. Furthermore, the local population, experiencing breathtaking economic and social change -- moving from a nomadic to a settled lifestyle in half a century or so -- is likely to experience confused and contradictory aspirations as it seeks to adapt to the new circumstances, as evident for example in the struggle to accommodate tribal ways and values to an urban existence. Some local ideas and wishes may likely conflict with those of outside authorities with respect to measures deemed necessary to conservation.

After reading this paper, a reviewer for the *Journal of Ethnobiology and Ethnomedicine *commented "by the end of the paper I was confused about whether the Reserve had actually been implemented yet and whether there was any functioning system of co-management". This is exactly the point: there is and there is not, depending on your viewpoint. Any visitor to the Al Reem Reserve will see no difference between it and the surrounding region, not even a notice telling you that the Reserve exists. Local management is failing to meet international expectations, which paradoxically espouse current bio-cultural diversity views of conservation and local participation. Ignorance of one another's' standpoint on the Al-Reem Reserve is mutual between locals and outsiders. We have to enquire further into what conservation comprises in other cultural contexts and the implications of different views [66,67,104]. This may strike ecologists as perverse with their universal assumptions about all environments being subject to the same biological laws. But anything less is unlikely to operationalise the assumptions of co-management drawing on bio-cultural diversity. The alternative is back to the imposition of international, largely Western, notions of conservation featuring human exclusion zones, if politically viable, with all the problems they entail.

## Competing interests

The authors declare that they have no competing interests.

## Authors' contributions

PS planned and managed the research; he supervised data analysis and wrote the paper. AAA facilitated field work and assisted with translation. AKAAH helped with data collection and assisted with translation. All authors read and approved the final manuscript.

## Appendix: Endnotes

^1 ^The Ministry of Municipal and Agriculture Affairs, Department of Animal Resources.

^2 ^The village name Khouzan, for instance, derives from *kazenah *'to hold, keep, reserve', after a small depression in the local terrain where water collects and Umm Al Qahab likewise comes from the slightly undulating surface, supplying hollows where water collects after rainfall. The name Abu Sidrah 'father Sidrah' comes from the Sidrah tree (*Ziziphus *spp.), which grows prolifically adjacent to water sources, and Refaiq may be a corruption of *refeeq *'friend', signifying a place where one found a welcome adjacent to a well.

^3 ^People own water bowsers and receive payment from the Government for transporting water to their villages.

^4 ^The UNESCO Nomination File 2007:30 makes the same point. In Oman the use of increasingly salty groundwater has resulted in large areas of land becoming sterile.

^5 ^Either married Qatari men who own registered animals or unmarried men over 30 years in age; those married to Qatari women can also apply, claiming the right in their wife's name (their children may subsequently claim permits in their own names if Qatar residents).

^6 ^Five have no licensed stock and are recreational camps only (marked *). The Ministry has Qura Abu Raghed camp incorrectly listed in Jemailiya Municipality.

^7 ^Table 2 lists Qura Abu Raghed camp in Jemailiya Municipality, as in the Ministry records.

^8 ^Data in this and the following table (of March 2009) kindly supplied by the Department of Animal Resources (Ministry of Municipal and Agriculture Affairs).

^9 ^Five locations had two separate stock licenses in different names and treated as separate camps.

^10 ^Staff at the Ministry talked about people messing around with registration numbers on tags, albeit such practices are illegal. (It is not possible to tamper with camel registration as this involves a micro-chip inserted into the animal's neck.)

^11 ^According to these animal totals in the Al Reem region two years previously (April 2007) were: 947 camels, 181 cattle, 9073 sheep, 8 horses and 17 donkeys. (Ministry of Environment briefing notes entitled 'Al-Reem Reserve: Man & Biosphere Reserve' kindly supplied by Khalid Helal Al-Enzi.)

^12 ^According to the Ministry of Environment briefing notes on the Reserve, there are seventy-four farms in the region covering 1,839 hectares.

^13 ^Some families continue to herd a few goats, sheep and camels to supply themselves, as previously, with meat and milk, albeit augmenting what they consume with food purchased using cash incomes. Others keep larger numbers of animals, notably goats and sheep, and to a lesser extent cattle, to supply the local Qatari market with meat (e.g. via the large wholesale market off Salwa Road in Doha), in addition to providing their families' needs.

^14 ^They do so seasonally, milking largely during the winter months, for although a camel will lactate for one year, they say that they milk for only some of this time, ceasing when the cow is pregnant again.

^15 ^Racing camels are mostly kept in stables adjacent to the Al Shahaniya racing track and not in remote places such as Al Reem camps.

^16 ^The identity issue is a complex one, for as Cole page 380 [83] points out, prized camels "had genealogies and histories that were intertwined with those of their owners".

^17 ^Webster page 492 [53] makes similar recommendations for the Jubail Reserve on the Saudi Arabian Gulf.

^18 ^Obscure wording of the question in Arabic may partly explain why so few respondents provided definitions.

^19 ^Meteorological data supplied by Department of Meteorology, Qatar Civil Aviation Authority.

^20 ^Note that some of the responses suggest that respondents did not understand what an exclosure is, confusing it with enclosure. See [105] for discussion of enclosures to protect wildlife such as oryz.

^21 ^Hunting is an important activity for some men in the Al Reem region, not for what it brings to the cooking pot, as previously, but again as an aspect of their Arab identity, particularly the use of falcons; 20 out of the 23 survey respondents said that they hunt with falcons, some also use dogs and guns. It is a unique aspect of their desert cultural heritage - whereas people around the world hunt with guns and dogs, only in southwest Asia do they regularly use falcons. These birds, like camels, can change hands for large sums (e.g. prices quoted by falcon sellers at Souq Waqif run to hundreds of thousands of Riyals). The houbara bustard (*Chlamydotis undulata macqueenii*) and cape hares [*Lepus capensis*] are favourite prey species.

^22 ^An increasingly popular market-informed approach to conservation is to advocate that people are remunerated for the ecosystem services of protected natural environments. But there is little mileage in this approach in desert contexts where - unlike forest watersheds - there is little vegetation to fix CO_2 _and release O_2_, and scant water catchment function and erosion protection. And national income from hydrocarbon revenues far exceeds any ecosystem service remuneration yet contemplated - so this market solution is unlikely to fly.

^23 ^According to one botanical study however, the passage of a single vehicle may benefit plant growth by creating a shallow indentation where rainwater collects [106] - although this is at odds with current opinion [107].

^24 ^When long-term camp permit-holders move camp, they should clear up the previous site as a condition for securing a new permit, and Ministry staff are supposed to go and check that they have done so properly when they reapply.

## Supplementary Material

Additional file 1 **Appendix I, Questionnaires**. Click here for file
