# Supplementary material for: Challenges to conservation: land use change and local participation in the Al Reem Biosphere Reserve, West Qatar
Source: J Ethnobiol Ethnomed. 2010 Oct 21;6:28. doi: 10.1186/1746-4269-6-28 (PMC2987904; doi:10.1186/1746-4269-6-28)
Supplement: Additional file 1 — Appendix I, Questionnaires. [file 1746-4269-6-28-S1.PDF]

Appendix I. The Survey Schedules.

دراسه مسجله لجمع معلومات عن الترتيبات الاجتماعيه والاقتصاديه

Survey schedule to collect data on social and economic arrangements

تفاصيل المجيب الاسم:<br>Respondent details: Name:	
الجنس<br>Sex:	ذكر/أنثى<br>M/F
العمر(تقريبي):<br>Age (estimated):	<15 16-25 25-45 46/60 >61
الجنسيه:<br>Nationality:	قطري /أخرى<br>Qatari/Other
اذا أخرى, ماهي؟<br>If other, give nationality:	
اذا قطري, ماهي اسم القبيله؟<br>If Qatari, give tribal affiliation:	
موقع السكن في منطقة الريم:<br>Location of residence in Al Reem region:	اسم المنطقه:<br>Place name:
موقع السكن في الخريطه مع الرقم:<br>Locate residence on map with number	رقم السكن:<br>House/Tent No:
مانوع الاقامه؟<br>What sort of resident are you?:	دائم/ مؤقت<br>permanent/temporary
كم هي فترة السكن في منطقة الريم؟<br>How long have you lived in Al Reem region?	
	ملكية عقار , عامل , سائح , أخرى

السبب في التواجد في منطقة الريم:<br><br>Reason for being in Al Reem region:	(تفصيل)<br><br>own land contract labour other (specify)
ماهي مهنتك؟<br><br>What is you occupation?	
Do you own any animals?<br><br>هل تمتلك أي نوع من الحيوانات؟	Y/N<br>لا/نعم
If so, how many?<br><br>اذا كنت تمتلك فعلا، كم عدد كل من؟	Camels: الجمال:<br><br>Goats: الماعز:<br><br>Sheep: الأغنام:
Do you buy animal feed?<br><br>هل تشتري علف للحيوانات؟	Y/N<br>لا/نعم
If so, what & how much?<br><br>اذا كنت فعلا، مانوعها وكم عددها؟	
كم عدد الاشخاص الذين يعيشون في سكنك؟<br><br>How many people are there in your household?	

Household members details:

العمل<br>Job	العمر<br>Age	الجنسية<br>Nationality	عدد الخدم والعمال<br>Workers & housekeepers		الجنسية<br>Nationality	العمل<br>Job	الحالة التعليمية<br>Education	الحالة الزواجية<br>Marital status	Relationship with household head<br>F M B S GP other kin (specify), friend(s)<br>أب , أم , أخ , أخت , أجداد , أقارب أخرين (تفصيل), أصدقاء(	Age العمر	Sexالجنس		الرقم المسلسل
			Fاناث	Mذكور							Fأنثى	ذكر M	
													.1
													.2
													.3

													.4
													.5
													.6
													.7
													.8

دراسة لمعرفة آراء المجتمع المحلي عن محمية الريم

Survey schedule to gauge local knowledge and opinions of Al Reem MAB Reserve

تفاصيل المجيب الاسم:<br>**Respondent details:** Name:	
الجنس<br>Sex:	ذكر/أنثى<br>M/F
العمر(تقريبي):<br>Age (estimated):	<15 16-25 25-45 46/60 >61
الجنسيه:<br>Nationality:	قطري /أخرى<br>Qatari/Other
اذا أخرى, ماهي؟<br>If other, give nationality:	
موقع السكن في منطقة الريم:<br>Residence in Al Reem region:	دائم/ مؤقت<br>permanent/temporary
السبب في التواجد في منطقة الريم:<br>Reason for being in Al Reem region:	ملكية عقار , عامل , سائح , أخرى<br>own land contract labour<br>tourist other ( )
ماهي مهنتك؟<br>What is you occupation?	
ماهو مستواك التعليمي؟<br>What grade did you reach in school?	
الاسئله:<br>**Questions:**	
هل تعلم ان منطقة الريم هي محميه؟<br>Do you know that Al Reem is MAB Reserve?	نعم/ لا<br>Y/N
(ملاحظه: (اذا كانت الاجابة لا) يجب اخبار المبحوث بأختصار عن محمية الريم ؟)<br>*[Note: if N, it will be necessary to tell respondent briefly what a MAB Reserve is.]*	

هل تعرف ماهي المحمية من قبل؟<br><br>Do you know what conservation is?	نعم/ لا<br><br>Y/N
What is conservation and why it is necessary?<br><br>هل تعتقد انها مهمه؟	
(ملاحظه: : (اذا كانت الاجابة لا) يجب اخبار المبحوث بأختصار عن ماهي المحافظه؟)<br><br>*[Note: if N, it will be necessary to tell respondent briefly what conservation is.]*	
هل تعتقد أن الريم بكونها محميه شي جيد؟<br><br>Do you think that Al Reem being a MAB Reserve is a good thing?	نعم/ لا<br><br>Y/N
هل تعلم عن الخطه لتدبير التدخل لتحسين حالة الموارد الطبيعيه و ارجاع الحياة البريه؟<br><br>Do you know about planned management interventions to improve the state of natural resources and bring back wildlife.	نعم/ لا<br><br>Y/N
(ملاحظه: : (اذا كانت الاجابة لا) يجب اخبار المبحوث بأختصار عن ماهي الخطه التدبير؟)<br><br>*[Note: if N, it will be necessary to tell respondent briefly about management plans.]*	
ماذا تعتقد عن الاربع أهداف الرئيسيه لتدبير التدخل؟<br><br>What do you think of the four main proposed management interventions? They are:	
أنشاء مناطق مغلقه (محاطه بسياج صغيرلمنع الحيوانات من الدخول)<br><br>Establishment of exclosures (small fenced areas to keep animals out and increase seed bank)	هل تعتقد بأن هذا الشيء جيد/ سيء؟ ولماذا؟<br><br>Do you think this is a good/bad thing. Why?
اعداة بناء المجتمع المحلي استنادا على نظام المحمية<br><br>Re-establishment of community based *hima* grazing system	هل تعتقد بأن هذا الشيء جيد/ سيء؟ ولماذا؟<br><br>Do you think this is a good/bad thing. Why?
التحكم في دخول لمركبات<br><br>Control of vehicular access	هل تعتقد بأن هذا الشيء جيد/ سيء؟ ولماذا؟<br><br>Do you think this is a good/bad thing. Why?
	هل تعتقد بأن هذا الشيء جيد/ سيء؟ ولماذا؟<br><br>Do you think this is a good/bad

منع الصياده المحليه<br><br>Prohibition of hunting locally	thing. Why?
Do you hunt?<br><br>هل تقوم بالصيد؟	Y/N<br>لا/نعم
If so, how often?<br><br>اذا كنت فعلا، كم مره تذهب للصيد	days per year<br><br>يوما في السنه
How do you hunt?<br><br>كيف تقوم بالصيد ؟	Falcon: الصقور:<br><br>Dog: الكلاب:<br><br>Other: آخر:
Do you collect truffles?<br><br>هل تجمع الفجع؟	Y/N<br>لا/نعم
Is the area good for truffles?<br><br>هل تعتبر المنطقة جيدة للفجع؟	Y/N<br>لا/نعم
Are there specific sites that are exceptionally good in Al Reem?<br><br>هل توجد بالمنطقة مواقع متميزة للفجع؟	If so where?
